# Differential metabolic profiles associated to movement behaviour of stream-resident brown trout (*Salmo trutta*)

**DOI:** 10.1371/journal.pone.0181697

**Published:** 2017-07-27

**Authors:** Neus Oromi, Mariona Jové, Mariona Pascual-Pons, Jose Luis Royo, Rafel Rocaspana, Enric Aparicio, Reinald Pamplona, Antoni Palau, Delfi Sanuy, Joan Fibla, Manuel Portero-Otin

**Affiliations:** 1 Animal Science Department, ETSEA, University of Lleida, Lleida, Catalonia, Spain; 2 Institute of Biomedical Research of Lleida (IRBLleida), University of Lleida, Lleida, Spain; 3 Area of Biochemistry and Molecular Biology, School of Medicine, University of Malaga, Málaga, Spain; 4 Gesna Estudis Ambientals, S.L., Linyola, Lleida, Catalonia, Spain; 5 GRECO, Institute of Aquatic Ecology, University of Girona, Catalonia, Spain; 6 Environment and Soil Sciences Department, ETSEA, University of Lleida, Lleida, Spain; National Cheng Kung University, TAIWAN

## Abstract

The mechanisms that can contribute in the fish movement strategies and the associated behaviour can be complex and related to the physiology, genetic and ecology of each species. In the case of the brown trout (*Salmo trutta*), in recent research works, individual differences in mobility have been observed in a population living in a high mountain river reach (Pyrenees, NE Spain). The population is mostly sedentary but a small percentage of individuals exhibit a mobile behavior, mainly upstream movements. Metabolomics can reflect changes in the physiological process and can determine different profiles depending on behaviour. Here, a non-targeted metabolomics approach was used to find possible changes in the blood metabolomic profile of *S*. *trutta* related to its movement behaviour, using a minimally invasive sampling. Results showed a differentiation in the metabolomic profiles of the trouts and different level concentrations of some metabolites (e.g. cortisol) according to the home range classification (pattern of movements: sedentary or mobile). The change in metabolomic profiles can generally occur during the upstream movement and probably reflects the changes in metabolite profile from the non-mobile season to mobile season. This study reveals the contribution of the metabolomic analyses to better understand the behaviour of organisms.

## Introduction

Migration phenomenon allows the distribution of animals across space and time and is basic to understand the ecological and evolutionary processes. Migratory strategies vary between and within species, and a common form of migration is known as partial migration [1]. This occurs when just a fraction of individuals from a population migrate while the others remain residents [1,2]. Partial migration is well documented in fishes, especially in salmonids [2], but salmonids exhibit large differences in behaviour between populations of the same species, as well as within populations, and even among siblings [3,4]. The mechanisms that can explain the mobile behaviour are complex, playing the life-history type an important role on development and behaviour [5].

Some salmonids migrate from the sea to the rivers (anadromy) or along the same river (potamodromy) depending on the species and the access to their target [3,6]. In the case of brown trout, it shows a great plasticity in its migratory behaviour and exhibits different life history tactics [7]. For example, inhabiting in temperate coastal streams, or streams connected to larger lakes (lentic environments) generally migrate, while many inland populations do [7]. Recent studies using mark-recapture methods and telemetry have shown that most salmonids are relatively sedentary, including the brown trout, with a limited movement associated with spatial competition [8–10]. In a population of *S*. *trutta* was shown that a high proportion of trouts remained in the same part of a river reach 800 m length (acting as its home range) during the whole year [8]. Upstream movements, around only 100 m, were also reported by Höjesjö et al. [9] in *S*. *trutta* in a Swedish river. However, little is known about the pattern of movements in the brown trout of the Mediterranean region, where all the populations are stream-resident. Some data from upper Pyrenean streams [11] showed a 60–80% of recaptured fish within 100 m of the sites where were originality marked, and only a 2–6% of individuals with maximum displacement distance over 500 m. Vera et al. [12] also working in a Pyrenean river of north-eastern Spain, showed similar conclusions. These results are consistent with the behaviour observed in other rivers [13,14]. Although stream-resident *S*. *trutta* seems to be mainly sedentary, a small proportion of individuals exhibit a mobile behaviour [11,12,15]. The mechanisms underneath this behaviour can be complex and probably related to the physiology, genetic and ecology of the brown trout.

As a part of a global project that aim to assess a physiological, genetic and ecological approach to understand individual differences in movement behaviour of *S*. *trutta* in Mediterranean streams, in the present study we searched for a metabolomic profile associated with the pattern of movements in *S*. *trutta*. In fishes, metabolomic techniques have mainly used in the effects of chemical exposure, in physiology and development, and also as application in human toxicology or fish as foodstuffs [16]. Metabolomics can reflect changes in the physiological process and can determine different profiles depending on behaviour (e.g. in the migration of Sockoye Salmon [17]). Therefore, changes in the metabolite concentrations can be key to differentiate individual movement behaviour- the sedentary and the mobile- observed in *S*. *trutta*. Here, a non-targeted metabolomic method was used to find possible changes in the blood metabolomic profile of *S*. *trutta* related to its movement behaviour. On the one hand, plasma metabolites can inform about the energetic status of individuals. On the other hand, the metabolic patterns should be differentiated depending on whether the individual has moved upstream or if, on the contrary, it exhibits a sedentary behaviour. In addition, we evaluated the metabolites associated with movement that can define the upstream phenotype in brown trout from Pyrenean streams. In this sense, the metabolomic profile could differentiate the small percentage of individuals with upstream movements. In fact, movements and migration is considered as one of the most demanding and physiologically challenging phases of salmon life history and represents a complex interplay between physiology and behaviour [18].

## Material and methods

### Ethics statement

Permissions for electrofishing and capture of *S*. *trutta* individuals, was approved by the competent authorities: Departament de Medi Ambient i Habitatge de la Generalitat de Catalunya (current Departament d'Agricultura, Ramaderia, Pesca, Alimentació i Medi Natural) (SF/602) of the regional authorities of Catalonia.

### Study area

Flamisell River is a small headwater tributary to the Noguera Pallaresa River (Ebro Basin, Catalonia, NE of Iberian Peninsula), which flows from the Pyrenees to the south for 31 km. The study river section was situated from la Plana de Montros (42°23'08.8"N, 0°57'39.8"E) to 3.5 km upstream. The reach has a mean slope of 53.3 m/km and channel morphologies consist primarily of pool-run-riffle sequences under a forest canopy. The hydrological regime is nival, with minimum flows in summer and winter, and major peaks usually in spring after snowmelt (Ebro Water Authority; http://www.chebro.es). Brown trout is the only fish species present and the population belongs to Mediterranean lineage [19].

### Fish movement estimation

A total of 997 brown trout ranging between 80 to 310 mm in fork length (mean 143.2 mm ± 36.0 S.D.) were captured by backpack electrofishing in April of 2013. Fish were anaesthetised with MS-222 (tricaine methanesulfonate) and tagged with HDX PIT tags (Oregon RFID, Portland, OR, USA) before being released at the same point of capture. Locations of PIT-tagged brown trout were determined with a portable PIT tag antenna [20]. PIT detection surveys were performed during 7 sampling seasons in 2013 (July, August, September, October, November, December) and 2014 (February). The brown trout population showed overall limited mobility although some individuals exhibit long-range movements. Considering all movement data, the 76.8% of the recorded movements between sampling seasons were less than 20 m, and only 3.6% of the recorded movements were higher than 200 m. The histogram distribution of the distance between the upstream-most and downstream-most position recorded for each individual (i.e. home range) was used to classify fish as sedentary (home range < 50 m) or mobile (home range > 50 m). Thus, we considered that sedentary individuals spend most of their lives in short reaches of stream less than 50 m, according to Rodriguez [21]. These two groups were subsequently used to test for differences in metabolite concentration.

### Sampling method

During the sampling season of November, blood samples were obtained from 28 trouts captured using electrofishing across the studied river section. This sampling season were selected because is the beginning of the reproductive period in which the individuals could make the largest upstream movements. We also chose a single extraction point to avoid animal suffering and minimize stress to affected individuals. The characteristics of each individual (i.e. home range, sex and fork length) are shown in Table 1. All the fish followed the same blood extraction protocol: 1) individuals were captured using electrofishing and immediately placed into a water tank of 5 L with MS-222 anaesthetic (25 mg/L), 2) after 10 min of anaesthesia, the blood was taken laterally from the caudal vein (50–100 μL) using a heparinized syringe and tubes to prevent clotting, 3) subsequently, blood samples were centrifuged for 5 min (2000 g) to precipitate the red blood cells and the plasma was transferred into a fresh Eppendorf tube and frozen in a liquid nitrogen tank, 4) after blood extraction, individuals were placed in a tank and released in the same site (or section) in which was caught after recovering (10 min approximately). Note that all the manipulations were done in the proximities of the river to maximum avoid the disturbance of the individuals. No damage to the caudal fin was observed to result from the blood sampling. At follow up, individuals appeared undisturbed by this process.

**Table 1:** Characteristics of the 28 brown trout used in the metabolite analysis.

ID	Home range (m)	Fork length (mm)	Sex	Class
ST0925	5	138	f	SE
ST0619	6	188	f	SE
ST0017	8	147	f	SE
ST0057	10	105	m	SE
ST0676	10	155	m	SE
ST0683	12	126	f	SE
ST0582	13	159	m	SE
ST0070	14	123	m	SE
ST0915	14	183	f	SE
ST0018	16	145	f	SE
ST0036	17	159	m	SE
ST0544	23	160	m	SE
ST0979	26	199	f	SE
ST0876	29	119	m	SE
ST0799	51	105	m	MO
ST0916	89	194	m	MO
ST0062	98	161	f	MO
ST0538	118	236	f	MO
ST0740	178	195	m	MO
ST0836	226	188	f	MO
ST0204	228	170	m	MO
ST0896	251	127	m	MO
ST0856	645	128	f	MO
ST0820	712	146	f	MO
ST0702	823	159	m	MO
ST0512	1102	201	f	MO
ST0515	1255	165	f	MO
ST0129	1733	129	m	MO

m = male, f = female, SE = sedentary, MO = mobile

### Non-targeted metabolomics

Metabolites were analysed in the 28 individuals using 10 μL of plasma for each sample. Detailed protocol is available on protocol.io (10.17504/protocols.io.h88b9zw). Briefly, after plasma deproteinization using methanol, samples were submitted to liquid chromatography coupled to high resolution mass spectrometry, using a Q-TOF and detecting molecules positively ionized in full-scan electrospray mode from 100–3000 m/z, as indicated in [22]. Only common features (found in at least 75% of the samples of the same condition, in this case migration characteristics) were analyzed, correcting for individual bias. Metabolite annotation was performed by using the database PCDL from Agilent (Agilent Technologies), using both accurate masses and retention time identity with the ones present in the database obtained by injecting authentic standards in an identic chromatographic system according to previously published works [22].

### Statistical analyses

The MetaboAnalyst 3.0 [23] platform was used to analyse the potential influences of home range, sex and fork length in the metabolomic profile as well as the metabolic pathway impact. We filtered by interquartile range, log transformed metabolite intensity, and autoscaled them before further analyses. To explore the differences patterns of metabolic profiles between the sedentary and mobile trouts, multivariate statistical analysis were used, including principal component analysis (PCA) and partial least-squares discriminant analysis (PLSDA). For univariate statistics (correlation with home range and differentiation between mobile and sedentary individuals), we employed Benjamini-Hochberg correction for false discovery rate (FDR, p < 0.05), a common practice in non-targeted metabolomics[24].

To evaluate the possible association between the identified metabolites and the home range, the molecules with a significant correlation with the home range were selected, using a similar approach (FDR, p < 0.05). The metabolites with a significant correlation were used to perform an over-representation analysis for pathway mapping. This analysis tests if a selected group of compounds is represented more than expected by chance within the reference compound list (in this case *Danio rerio* reference metabolome)[25]. In addition, we used Interactome analyses performed by searching shared metabolites between different pathways (at least 1 metabolite and p < 0.05, employing Reactome as database and the CPFB platform (http://cpdb.molgen.mpg.de), using the same list of enriched metabolites[26].

## Results

### Metabolite analysis detection

The non-targeted approach allowed to detect 5734 different molecular features in plasma from *S*. *trutta* individuals. Although sex affected the metabolomic profile (S1 Table), either false discovery rate correction and/or inclusion of mobile status (2 way ANOVA, differential molecules due to migratory status: 227, due to sex: 0) demonstrated that sex was not a relevant variable in comparison to the mobility pattern. Similarly, size was not a major factor in comparison to the mobility pattern. Fork length showed a low number of metabolites correlated (163 with p <0.05, 0 corrected for FDR, S2 Table), in comparison with home range (see below).

**Fig 1:**
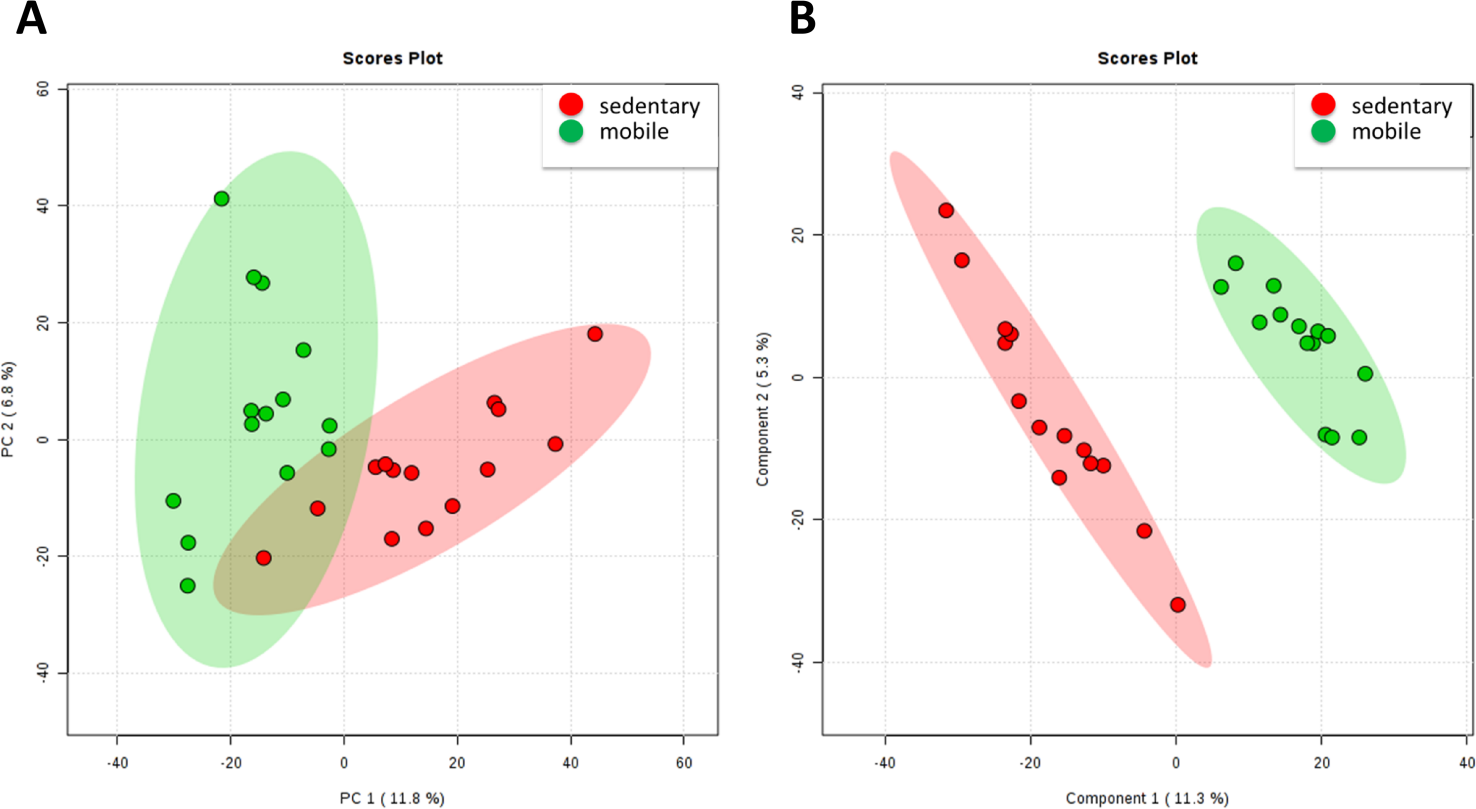
Multivariate analyses show the existence of plasma metabolomic patterns differing between sedentary and mobile individuals. Both at a priori model (PCA), shown in A and a posteriori model (Partial least square discriminant model, PLSDA) shown in B demonstrate that plasma metabolomic profiles differ between sedentary and mobile individuals.

**Fig 2:**
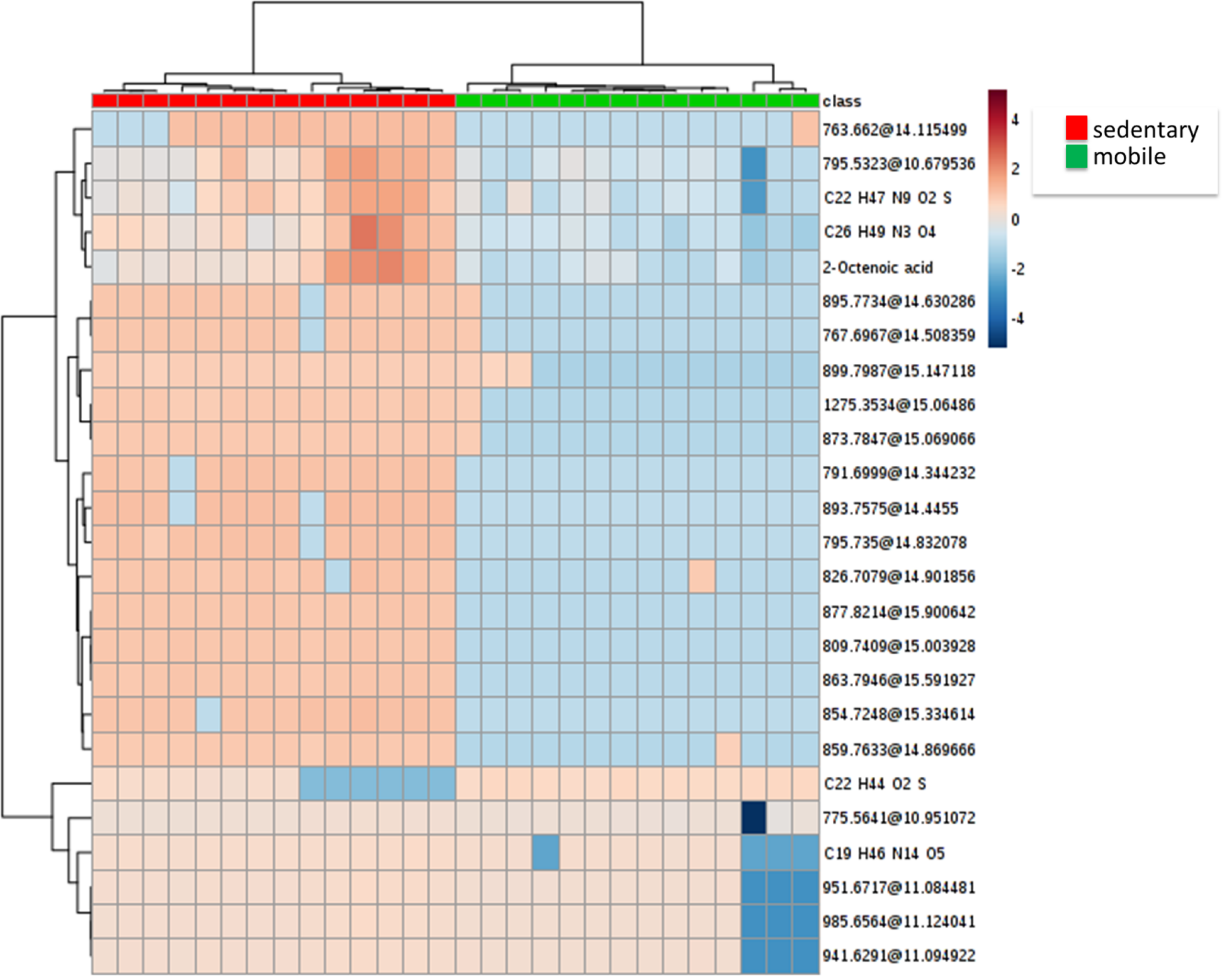
Heatmap shows significant clustering of individuals depending on movement: Sedentary (class colour red), mobile (class colour green) achieved by using plasma metabolomic profiles. The scale from −4 (blue) to 4 (red) represents this normalized abundance in arbitrary Unknown identities are represented as exact mass and retention time.

Multivariate analyses showed that 18.6% of variability could be explained by two component model using PCA, with a good clustering of the individuals according to home range classification (sedentary and mobile, Fig 1A). Indeed, PLSDA showed a similar pattern of classification (Fig 1B), with a very high accuracy (95.4% for 1 component), suggesting that metabolomic profiles could help to differentiate sedentary from mobile trouts. Reinforcing this concept, hierarchical clustering analyses (Fig 2), only including the top 25 molecules with a high correlation with home range-differentiated both groups.

### Pathway analysis

Significant correlation between the metabolite concentration and the home range of trout was found in 15 annotated metabolites (Table 2), despite many others, unknown metabolites also correlated (719 with p < 0.05, 285 corrected for FDR, S3 Table for Spearman correlation).

**Table 2:** Metabolites correlating with home range of trout.

**Molecule**	**correlation**	**p-value**	**FDR**
Retinaldehyde	-0.954	<0.001	<0.001
2-Octenoic acid	-0.939	<0.001	<0.001
Diethyl Oxalpropionate	0.846	<0.001	<0.001
5b-dihydroprogesterone	-0.768	<0.001	<0.001
Estrone	-0.766	<0.001	<0.001
Cortexolone	-0.720	<0.001	<0.001
11a-hydroxyandrost-4-ene-3_17-dione	-0.695	<0.001	0.001
16b-Hydroxyestradiol	-0.683	<0.001	0.001
Methyl linolenate	-0.672	<0.001	0.002
D-Galactose	-0.655	<0.001	0.003
B-estradiol	-0.565	0.002	0.023
Cortisol 21-acetate	-0.554	0.002	0.026
Taurocholic acid	-0.554	0.002	0.026
N-Oleoyl-D-erythro-Sphingosine (C18:1 Ceramide)	-0.521	0.004	0.047
Phytosphingosine	0.516	0.005	0.049

P value after Pearson correlation analyses. FDR: False-discovery corrected p value after Benjamini-Hochberg correction.

These metabolites were used to perform an over-representation analysis for pathway mapping. This analysis test if a selected group of compounds is represented more than expected by chance within the reference compound list (in this case *Danio rerio* reference metabolome). This algorithm identified 6 different pathways (Table 3) with metabolites correlating with home range or movement.

**Table 3:** Pathways associated with home range.

**Pathway affected**	**Total metabolites in pathway**	**Expected Hits**	**Actual hits**	**p value**
Retinol metabolism	16	0.18	2	0.012
Steroid hormone biosynthesis	56	0.63	3	0.022
Glycosylphosphatidylinositol-anchor biosynthesis	13	0.15	1	0.013
Sphingolipid metabolism	21	0.24	1	0.022
Glycerophospholipid metabolism	28	0.31	1	0.027
Purine metabolism	66	0.74	1	0.05

Annotated metabolites correlating with home range were mapped against metabolic pathways. P- values shown are those derived from a hypergeometric test.

**Fig 3:**
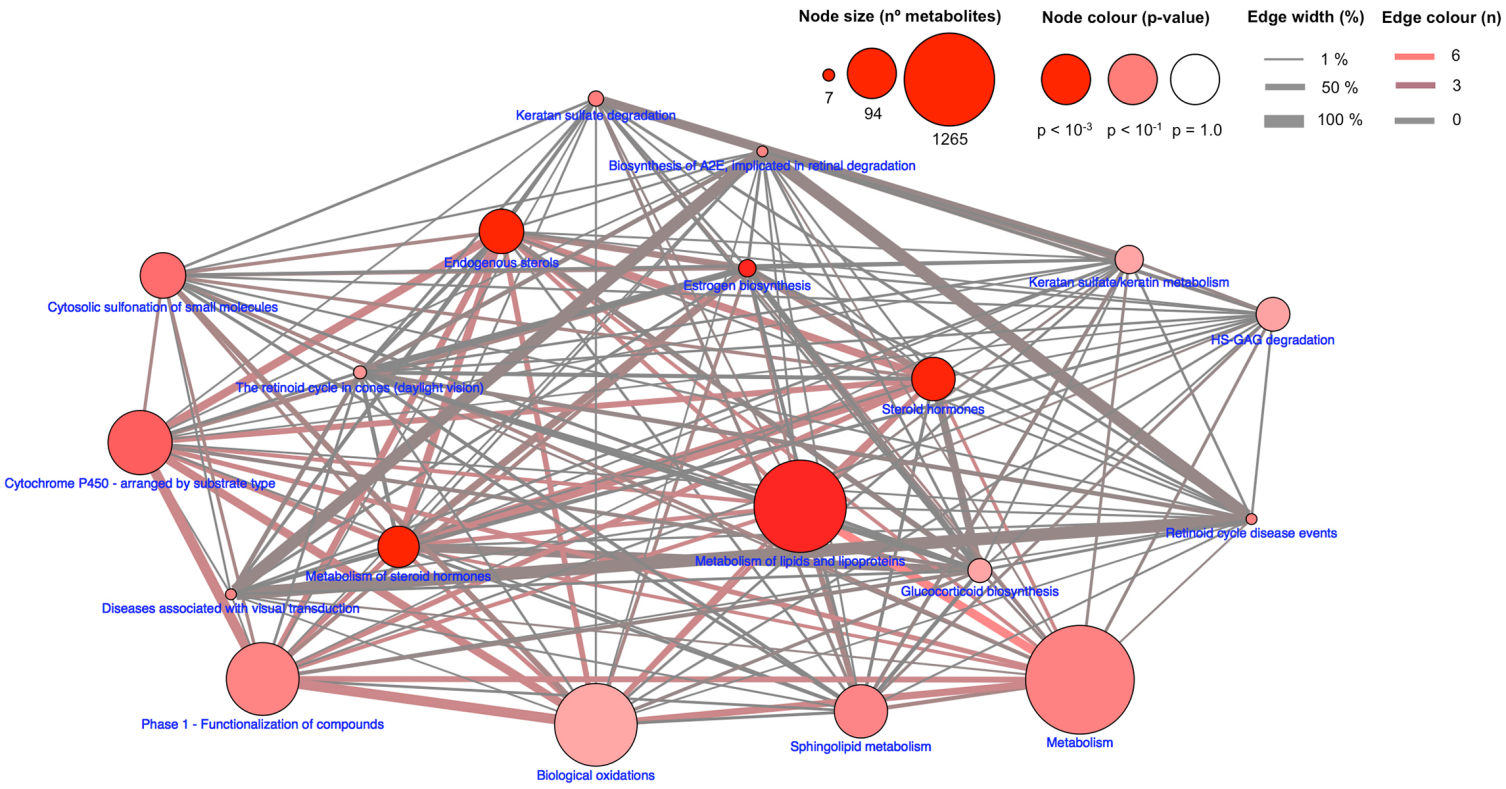
Interactomics of metabolites associated with home range. Metabolites were mapped to a pathway database (in this case Reactome), and nodes, representing pathways (identified by pathway name in the database), is proportional to the number of metabolites contained in the pathway. Node colour intensity is associated to hypergeometric test accounting number of metabolites associated with home range and those potentially present in the specific pathway (node), while as edge width represent the percentage of shared metabolites between pathways and edge colour indicate the number of metabolites associated with home range.

Interactome analyses (Fig 3), performed by the CPDB platform, which searches shared metabolites between different pathways (at least 1 metabolite and p<0.05, using Reactome as pathway database), indicates other nodes, such as *steroid hormones* and *lipid and lipoproteins* having an important role in the differentiation between mobile and sedentary individuals. Further, 4 pathways were related to retinal function.

## Discussion

The study of metabolite profiles may provide a closer link to functional physiological responses [27]. Concretely, the plasma metabolites are involved in energy production. For example, the concentration of triglycerides in plasma is a good indicator of bird health and reproductive success [28,29]. In Salmonids, a similar methodology was successfully used to study the reproductive energy investment in wild population of *Salmo trutta* using plasma metabolite variation [30]. In other study, the metabolite profiles in the Pacific salmon (*Onchorhychus nerka*) highly contrasted those at the spawning grounds resulting in two clear groupings [17]. In our study, 15 metabolites have been identified and related to the movements of *S*. *trutta*, using a non-targeted metabolomics approach. The metabolite profiles differentiated the individuals according to the home range classification (sedentary and mobile) and showed different level concentrations of some metabolites, which interact in different pathways. Considering that stream-resident *S*. *trutta* in the Flamisell river is mainly sedentary, the small proportion of individuals with mobile behaviour, clearly differentiated by the metabolic profiles, can have a relevant function in the population dynamic.

The variation in metabolomic profiles can generally occur during the upstream movement and probably reflects the changes from the non-mobile season (e.g. for feeding) to mobile season (e.g. reproduction) in the mobile individuals. In other Salmonid species, these changes can reach their peaks toward the end of migration [17,31]. Therefore, the differentiated metabolomic profiles found in our study, grouped the individuals that have performed a movement, which have been able to undergo physiological changes. In contrast, the sedentary individuals were represented in another group with different metabolomic profile. The different pathways that significantly affected home range allow to understand the physiological condition and changes that trouts can experience from the sedentary to mobile season, in the case of displacement. In addition, the study of each metabolite associated with home range allow to assess the possible particularities of the small percentage of individuals with upstream movements in comparison with those with the typical sedentary behaviour of Mediterranean *S*. *trutta* populations.

The steroid hormone biosynthesis pathway was significantly associated with home range. In vertebrates, steroids can play multiple roles in growth, stress response, reproduction and behaviour. However, the steroid dynamics is poorly understood and the studies are only limited to some aquaculture systems that show that fish manipulation affect cortisol and testosterone concentration in water [32]. In our study, the cortisol (Cortexolone and Cortisol 21-acetate) was negatively related to the home range or movement. In a wild population of salmonid, a subordinate individual may be subject to stress as a result of attacks and repeated threats of the most dominant individuals in the population competing for access to different resources (food, sexual activity and dominance of the territory). This social stress leads to a marked behavioural and physiological change in subordinates, who often show a generally less aggressive and locomotory activity, and also higher levels of plasma cortisol [16,33–35]. According to this argument, in our case, some dominant individuals can play the role of explorers (with upstream movements associated to the low levels of cortisol). However, other works have found elevated levels of cortisol in dominant aggressive fish [16]. On other hand, there were not differences in metabolite pattern between sexes. In the case of the cortisol, these results are similar to other studies in *S*. *trutta* [36] where the cortisol levels did not change between sexes during the year. A similar pattern was obtained for the estradiol, another steroid hormone that can directly affect energy metabolism [37]. Therefore, the change in cortisol and estradiol levels, as in most metabolites, can follow complex patterns linked to the fish behaviour. In fact, the mobile individuals could be also displaced trouts that are forced to move, upstream through the river, to find more suitable areas to improve feeding or reproduction-spawning opportunities. Their low levels of cortisol and estradiol could be derived from the effort after the upstream movement.

In our study, the metabolic pathways associated with home range, include the glycerophospholipid metabolism that could be related to the elevated levels of the stress hormone cortisol. The high levels of cortisol could have characteristic gene expression profiles in liver tissue, including metabolism of energy reserves via gluconeogenesis, glycolysis, and metabolism of amino acids and lipids [38,39]. On other hand, the pathway of glycosylphosphatidylinositol (GPI)-anchored is related to the nerve growth factor receptor (p75 NTR) and other coreceptors [40] that can be related to movement in mammals. But, no data on the functional properties and expression for this pathway have been published in fish [41]. In addition, the retinol metabolism pathway was associated with home range, with the retinaldehyde metabolite negatively correlated with movement. The main bioactive metabolites derived from vitamin A (or retinol) have different functions in development and adult physiology [42]. In fish, this metabolic pathway could affect growth and survival rate and performance, especially in larvae [43]. In addition, this pathway affect brain development, vision and learning and memory, which could be related to the changes between sedentary to mobile season [44]. Of note, these metabolites have been recently reviewed as key factors in circadian and seasonal changes, as well as with sleep function[45]. Other researchers have focused on the role of retinoids in behaviour and learning[46], key aspects in ethology.

Briefly, the results found in the present work reflect the importance of the study of the metabolomic profiles and its changes in order to identified individual behaviour. We acknowledge, as limitations of the present work that, additional studies are needed to investigate the changes in the metabolism of one individual, arising from movement-associated changes in locomotory behaviour. These studies include the determination of the expression pathway key genes or protein to demonstrate which could be the mechanism explaining changes in a given metabolite to ascertain if different expressions depended on behaviour or whether behavioural differences induced selective gene expressions. In another step, metabolite data could be complemented with proteins and transcriptomic data that related to the behaviour, can lead to the identification of multiple physiological biomarkers. Despite this fact, it's clear that metabolomic techniques are useful methodology, measuring changes rapidly and sensitively, for pattern-recognition analyses of biological samples. In addition, the minimally invasive sampling scheme presented here allow for the follow up of interesting species for conservation, with minimal risk for individuals, paving the way for scientific evaluation and evaluation of potential biomarkers related with migratory behaviour.

## Supporting information

S1 Table Metabolites correlating with sex. (DOCX)

S2 Table Metabolites correlating with fork length. (DOCX)

S3 Table Molecules correlating with home range. (DOCX)

S1 File Metabolite dataset. Set of metabolites raw abundances, integrated and distributed within individuals, with its individualized phenotypic characteristics.
(ZIP)

## References

[1] Chapman BB, Hulthén K, Brodersen J, Nilsson PA, Skov C, Hansson LA, et al Partial migration in fishes: Causes and consequences. J Fish Biol. 2012;81: 456–478. doi: 10.1111/j.1095-8649.2012.03342.x 22803720 10.1111/j.1095-8649.2012.03342.x

[2] Jonsson B, Jonsson N. Partial migration: niche shift versus sexual maturation in fishes. Rev Fish Biol Fish. 1993;3: 348–365.

[3] Jonsson B, Jonsson N. Ecology of Atlantic Salmon and Brown Trout—Habitat as a template for life histories. Fish and Fisheries Series. 2011.

[4] Klemetsen A, Amundsen P-A, Dempson JB, Jonsson B, Jonsson N, O’Connell MF, et al Atlantic salmon *Salmo salar* L., brown trout *Salmo trutta* L. and Arctic charr *Salvelinus alpinus* (L.): a review of aspects of their life histories. Ecol Freshw Fish. 2003;12: 1–59.

[5] Giger T, Excoffier L, Day PJR, Champigneulle A, Hansen MM, Powell R, et al Life history shapes gene expression in salmonids. Curr Biol. 2006;16. 10.1016/j.cub.2006.03.053 16631571

[6] Pavlov DS, Savvaitova K. On the problem of ratio of anadromy and residence in salmonids (Salmonidae). J Ichthyol. 2008;48: 778–791.

[7] Lucas MC, Baras E, Thom TJ, Duncan A, Slavík O. Migration of Freshwater Fishes. Oxford, UK: Wiley Online Library; 2001.

[8] Knouft JH, Spotila JR. Assessment of movements of resident stream brown trout, *Salmo trutta* L., among contiguous sections of stream. Ecol Freshw Fish. 2002;11: 85–92.

[9] Höjesjö J, Økland F, Sundström LF, Pettersson J, Johnsson JI. Movement and home range in relation to dominance; a telemetry study on brown trout *Salmo trutta*. J Fish Biol. 2007;70: 257–268.

[10] Lobón-Cerviá J. Why, when and how do fish populations decline, collapse and recover? the example of brown trout (*Salmo trutta*) in Rio Chaballos (northwestern Spain). Freshw Biol. 2009;54: 1149–1162.

[11] Sostoa A, Nadal J, Casals F, Aparicio E, Vargas MJ, Olmo JM, et al. Caudales ecológicos. Memoria final del Proyecto PIE 121.043 FECSA-UNESA. Documento Inédito, Barcelona. 2015.

[12] Vera M, Sanz N, Hansen MM, Almodóvar A, García-Marín JL. Population and family structure of brown trout, *Salmo trutta*, in a Mediterranean stream. Mar Freshw Res. 2010;61: 676–685.

[13] Cresswell RC. Post-stocking movements and recapture of hatchery-reared trout released into flowing waters—a review. J Fish Biol. 1981;18: 429–442.

[14] Hesthagen TB, Johnsen BO. Survival and growth of summer and autumn stocked 0+ brown trout, *Salmo trutta* L., in a mountain lake. Aquac Fish Manag. 1989;20: 329–332.

[15] Rocaspana R, Aparicio E, Palau A. Análisis del uso, la eficiencia y la necesidad del paso de peces en el azud de Salinas (río Cinca, Huesca). Monografía Endesa. 2012; Madrid, 71.

[16] Øverli Ø, Winberg S, Damsård B, Jobling M. Food intake and spontaneous swimming activity in Arctic char (*Salvelinus alpinus*): role of brain serotonergic activity and social interactions. Can J Zool. 1998;76: 1366–1370.

[17] Benskin JP, Ikonomou MG, Liu J, Veldhoen N, Dubetz C, Helbing CC, et al Distinctive metabolite profiles in in-migrating Sockeye salmon suggest sex-linked endocrine perturbation. Environ Sci Technol. 2014;48: 11670–11678. doi: 10.1021/es503266x 25198612 10.1021/es503266x

[18] Miller KM, Schulze AD, Ginther N, Li S, Patterson DA, Farrell AP, et al Salmon spawning migration: Metabolic shifts and environmental triggers. Comp Biochem Physiol—Part D Genomics Proteomics. 2009;4: 75–89. doi: 10.1016/j.cbd.2008.11.002 20403740 10.1016/j.cbd.2008.11.002

[19] Aparicio E, García-Berthou E, Araguas RM, Martínez P, García-Marín JL. Body pigmentation pattern to assess introgression by hatchery stocks in native *Salmo trutta* from Mediterranean streams. J Fish Biol. 2005;67: 931–949.

[20] Roussel JM, Haro A, Cunjak RA. Field test of a new method for tracking small fishes in shallow rivers using passive integrated transponder (PIT) technology. Can J Fish Aquat Sci. 2000;57: 1326–1329.

[21] Rodriguez MA. Restricted movement in stream fish: The paradigm is incomplete, not lost. Ecology. 2002;83: 1–13.

[22] Sana TR, Roark JC, Li X, Waddell K, Fischer SM. Molecular formula and METLIN personal metabolite database matching applied to the identification of compounds generated by LC/TOF-MS. J Biomol Tech. 2008;19: 258–266. 19137116 PMC2567134

[23] Xia J, Mandal R, Sinelnikov I V., Broadhurst D, Wishart DS. MetaboAnalyst 2.0-a comprehensive server for metabolomic data analysis. Nucleic Acids Res. 2012;40. 10.1093/nar/gks374 PMC3394314 22553367

[24] Connor SC, Hansen MK, Corner A, Smith RF, Ryan TE. Integration of metabolomics and transcriptomics data to aid biomarker discovery in type 2 diabetes. Mol Biosyst. 2010;6: 909 doi: 10.1039/b914182k 20567778 10.1039/b914182k

[25] Xia J, Wishart DS. MSEA: A web-based tool to identify biologically meaningful patterns in quantitative metabolomic data. Nucleic Acids Res. 2010;38. 10.1093/nar/gkq329 PMC2896187 20457745

[26] Herwig R, Hardt C, Lienhard M, Kamburov A. Analyzing and interpreting genome data at the network level with ConsensusPathDB. Nat Protoc. 2016;11: 1889–1907. doi: 10.1038/nprot.2016.117 27606777 10.1038/nprot.2016.117

[27] Rochfort S. Metabolomics reviewed: A new “omics” platform technology for systems biology and implications for natural products research. Journal of Natural Products. 2005 pp. 1813–1820. doi: 10.1021/np050255w 16378385 10.1021/np050255w

[28] Merila J, Svensson E. Fat reserves and health state in migrant *Goldcrest Regulus regulus*. Funct Ecol. 1995;9: 842–848.

[29] Masello JF, Quillfeldt P. Are haematological parameters related to body condition, ornamentation and breeding success in wild burrowing parrots *Cyanoliseus patagonus*? J Avian Biol. 2004;35: 445–454.

[30] Gauthey Z, Freychet M, Manicki A, Herman A, Lepais O, Panserat S, et al The concentration of plasma metabolites varies throughout reproduction and affects offspring number in wild brown trout (*Salmo trutta*). Comp Biochem Physiol -Part A Mol Integr Physiol. 2015;184: 90–96. 10.1016/j.cbpa.2015.01.025 25666363

[31] Cook K V., McConnachie SH, Gilmour KM, Hinch SG, Cooke SJ. Fitness and behavioral correlates of pre-stress and stress-induced plasma cortisol titers in pink salmon (*Oncorhynchus gorbuscha*) upon arrival at spawning grounds. Horm Behav. 2011;60: 489–497. doi: 10.1016/j.yhbeh.2011.07.017 21839080 10.1016/j.yhbeh.2011.07.017

[32] Twardek WM, Elvidge CK, Wilson ADM, Algera DA, Zolderdo AJ, Lougheed SC, et al Do protected areas mitigate the effects of fisheries-induced evolution on parental care behaviour of a teleost fish? Aquat Conserv Mar Freshw Ecosyst. 2017; doi: 10.1002/aqc.2718

[33] Denight ML, Ward JA. Relationship of chin spot size to dominance in the black-chinned mouthbrooding cichlid fish (*Sarotherodon melanotheron*). Anim Behav. 1982;30: 1099–1104.

[34] O’Connor KI, Metcalfe NB, Taylor AC. Does darkening signal submission in territorial contests between juvenile Atlantic salmon, *Salmo salar*? Anim Behav. 1999;58: 1269–1276. doi: 10.1006/anbe.1999.1260 10600149 10.1006/anbe.1999.1260

[35] Winberg S, Nilsson GE. Time course of changes in brain serotonergic activity and brain tryptophan levels in dominant and subordinate juvenile Artic charr. J Exp Biol. 1993;179: 181–195.

[36] Fregeneda-Grandes JM, Hernańdez-Navarro S, Fernandez-Coppel IA, Correa-Guimaraes A, Ruiź-Potosme N, Navas-Gracia LM, et al Seasonal and sex-related variations in serum steroid hormone levels in wild and farmed brown trout *Salmo trutta* L. in the north-west of Spain. J Water Health. 2013;11: 720–728. doi: 10.2166/wh.2013.086 24334846 10.2166/wh.2013.086

[37] Norris DO, Hobbs SL. The HPA axis and functions of corticosteroids in fishes In: Reinecke M., Zaccone G., Kapoor BG, editor. Fish Endocrinology. Enfield, New Hampshire: Science Publishers; 2006 pp. 721–766.

[38] Aluru N, Vijayan MM. Stress transcriptomics in fish: A role for genomic cortisol signaling. Gen Comp Endocrinol. 2009;164: 142–150. doi: 10.1016/j.ygcen.2009.03.020 19341738 10.1016/j.ygcen.2009.03.020

[39] Hook SE, Kroon FJ, Metcalfe S, Greenfield PA, Moncuquet P, McGrath A, et al Global transcriptomic profiling in barramundi *Lates calcarcifer* from rivers impacted by differing agricultural land-uses. Environ Toxicol Chem. 2016; doi: 10.1002/etc.3505 27219023 10.1002/etc.3505

[40] Giger RJ, Venkatesh K, Chivatakarn O, Raiker SJ, Robak L, Hofer T, et al Mechanisms of CNS myelin inhibition: evidence for distinct and neuronal cell type specific receptor systems. Restor Neurol Neurosci. 2008;26: 97–115. 18820405 PMC7259427

[41] Lang DM, Romero-Alemán M del M, Dobson B, Santos E, Monzón-Mayor M. Nogo-A does not inhibit retinal axon regeneration in the lizard *Gallotia galloti*. J Comp Neurol. 2016;525: 936–954. doi: 10.1002/cne.24112 27616630 10.1002/cne.24112

[42] Eroglu A, Harrison EH. Carotenoid metabolism in mammals, including man: formation, occurrence, and function of apocarotenoids. J Lipid Res. 2013;54: 1719–30. doi: 10.1194/jlr.R039537 23667178 10.1194/jlr.R039537 PMC3679377

[43] Boglino A, Ponce M, Cousin X, Gisbert E, Manchado M. Transcriptional regulation of genes involved in retinoic acid metabolism in *Senegalese sole* larvae. Comp Biochem Physiol Part—B Biochem Mol Biol. 2017;203: 35–46. 10.1016/j.cbpb.2016.08.007 27619487

[44] Aubin-Horth N, Letcher BH, Hofmann HA. Gene-expression signatures of Atlantic salmon’s plastic life cycle. Gen Comp Endocrinol. 2009;163: 278–284. doi: 10.1016/j.ygcen.2009.04.021 19401203 10.1016/j.ygcen.2009.04.021 PMC2706306

[45] Ransom J, Morgan PJ, McCaffery PJ, Stoney PN. The rhythm of retinoids in the brain. Journal of Neurochemistry. 2014 pp. 366–376 doi: 10.1111/jnc.12620 24266881 10.1111/jnc.12620 PMC4283048

[46] Olson CR, Mello C V. Significance of vitamin A to brain function, behavior and learning. Mol Nutr Food Res. 2010;54: 489–95. doi: 10.1002/mnfr.200900246 20077419 10.1002/mnfr.200900246 PMC3169332

