# Supplementary material for: Differential metabolic profiles associated to movement behaviour of stream-resident brown trout (Salmo trutta)
Source: PLoS One. 2017 Jul 27;12(7):e0181697. doi: 10.1371/journal.pone.0181697 (PMC5531495; doi:10.1371/journal.pone.0181697)
Supplement: S1 Table — (DOCX) [file pone.0181697.s001.docx]

**S1 Table. Metabolites correlating with sex.**

| **Molecule*** | **p-value** | **-LOG10(p)** | **FDR** |
| --- | --- | --- | --- |
| trans-Dehydroandrosterone + 9.5154 | 0.000 | 37.462 | 0.617 |
| C28 H43 N3 S | 0.001 | 29.105 | 0.679 |
| 1592.5791@13.177142 | 0.001 | 2.885 | 0.679 |
| 775.5523@13.120143 | 0.001 | 28.847 | 0.679 |
| 758.2253@13.449889 | 0.004 | 2.402 | 0.679 |
| C20 H41 N5 O8 | 0.004 | 23.774 | 0.679 |
| C30 H33 Cl O8 | 0.007 | 21.796 | 0.679 |
| 1154.6287@7.3113337 | 0.007 | 21.679 | 0.679 |
| C12 H18 N4 S | 0.007 | 21.578 | 0.679 |
| C19 H N O7 S5 | 0.009 | 20.586 | 0.679 |
| C39 H65 Cl N2 | 0.011 | 19.624 | 0.679 |
| C21 H41 Cl O2 S | 0.011 | 1.948 | 0.679 |
| C36 H53 N3 S | 0.012 | 19.258 | 0.679 |
| C27 H52 O S4 | 0.012 | 19.055 | 0.679 |
| C23 H38 N2 O2 S | 0.013 | 18.986 | 0.679 |
| 1590.5853@13.133601 | 0.016 | 17.892 | 0.679 |
| 4-(dimethylamino)azobenzene n-oxide | 0.018 | 17.498 | 0.679 |
| C25 H42 N12 O | 0.018 | 17.402 | 0.679 |
| C33 H60 O5 S3 | 0.019 | 17.315 | 0.679 |
| C22 H3 N O15 S5 | 0.022 | 1.659 | 0.679 |
| C23 H49 N5 O8 | 0.022 | 16.507 | 0.679 |
| 1566.1656@13.352429 | 0.022 | 1.648 | 0.679 |
| 825.1564@8.936 | 0.025 | 16.093 | 0.679 |
| C35 H13 N7 O S | 0.027 | 15.677 | 0.679 |
| 982.2708@13.945287 | 0.027 | 15.646 | 0.679 |
| 831.5933@13.327541 | 0.028 | 15.573 | 0.679 |
| C25 H49 N O2 S2 | 0.030 | 15.233 | 0.679 |
| 508.8395@11.306143 | 0.031 | 15.124 | 0.679 |
| 598.6829@8.484143 | 0.031 | 15.095 | 0.679 |
| C10 H22 O3 | 0.031 | 15.081 | 0.679 |
| 1240.3904@13.713999 | 0.031 | 15.079 | 0.679 |
| C25 H40 N10 O4 | 0.031 | 15.077 | 0.679 |
| 1209.2931@13.0845 | 0.031 | 15.076 | 0.679 |
| 1223.8143@13.1235 | 0.031 | 15.074 | 0.679 |
| C39 H69 N O6 S | 0.032 | 14.898 | 0.679 |
| C23 H53 N23 O S | 0.033 | 14.814 | 0.679 |
| 683.519@7.3510003 | 0.034 | 14.674 | 0.679 |
| 853.5777@13.120381 | 0.035 | 14.615 | 0.679 |
| 857.605@13.391292 | 0.035 | 14.513 | 0.679 |
| 1531.0824@13.121857 | 0.037 | 14.304 | 0.679 |
| C28 H5 N3 O14 S | 0.038 | 14.233 | 0.679 |
| C31 H41 N3 O | 0.039 | 14.111 | 0.679 |
| C14 H37 N15 O3 | 0.040 | 13.985 | 0.679 |
| C41 H49 N3 O4 | 0.042 | 13.794 | 0.679 |
| C13 H16 O3 + 8.950473 | 0.042 | 13.754 | 0.679 |
| C38 H67 N S2 | 0.042 | 13.739 | 0.679 |
| C12 H28 N2 O3 S2 | 0.042 | 13.737 | 0.679 |
| C13 H37 N9 O2 S | 0.042 | 13.733 | 0.679 |
| C21 H49 Cl N18 O2 S | 0.042 | 13.726 | 0.679 |
| C36 H73 Cl N4 O2 S | 0.043 | 13.709 | 0.679 |
| 757.2257@13.450334 | 0.044 | 13.577 | 0.679 |
| C34 H55 Cl | 0.044 | 13.553 | 0.679 |
| C33 H36 Cl2 N8 S3 | 0.044 | 13.531 | 0.679 |
| C9 Cl3 N O3 S4 | 0.045 | 13.495 | 0.679 |
| 370.088@11.565685 | 0.045 | 13.449 | 0.679 |
| C30 H39 N19 O | 0.045 | 13.431 | 0.679 |
| C29 H57 N O4 S3 | 0.046 | 13.401 | 0.679 |
| C33 H71 N13 O4 S | 0.047 | 13.303 | 0.679 |
| C36 H65 N13 O3 | 0.048 | 13.224 | 0.679 |

P value after Student’s T test analyses. FDR: False-discovery corrected p value after Benjamini-Hochberg correction. *Differential ions presented are preliminary characterized by potential elemental formulae or, when it is not possible, characterized by m/z and retention time (in minutes) separated by the @ symbol.
