# Supplementary material for: Differential metabolic profiles associated to movement behaviour of stream-resident brown trout (Salmo trutta)
Source: PLoS One. 2017 Jul 27;12(7):e0181697. doi: 10.1371/journal.pone.0181697 (PMC5531495; doi:10.1371/journal.pone.0181697)
Supplement: S2 Table — (DOCX) [file pone.0181697.s002.docx]

**S2 Table. Molecules correlating with fork length.**

| **Molecule*** | correlation | p-value | FDR |
| --- | --- | --- | --- |
| C29 H33 N5 | -0.63808 | 0.00025901 | 0.44549 |
| 4-Pyridoxic acid | 0.62026 | 0.00043004 | 0.49311 |
| C17 H47 N11 O S2 | 0.58708 | 0.0010227 | 0.7163 |
| C19 H35 N9 O4 | -0.58636 | 0.0010411 | 0.7163 |
| C18 H41 Cl N4 O | 0.53425 | 0.0034064 | 0.73021 |
| C8 H17 N7 O | -0.52094 | 0.0044783 | 0.73021 |
| C20 H41 N5 O8 | -0.51438 | 0.0051037 | 0.73021 |
| C26 H43 N3 O S2 | -0.49352 | 0.0076123 | 0.73021 |
| C22 H29 N13 | -0.48683 | 0.0086091 | 0.73021 |
| C11 H24 Cl N3 O4 | 0.48391 | 0.0090774 | 0.73021 |
| 3-Isochromanone | -0.48158 | 0.0094668 | 0.73021 |
| C16 H34 N14 O4 | -0.47494 | 0.010653 | 0.73021 |
| C13 H28 N6 O | -0.47466 | 0.010704 | 0.73021 |
| C43 H26 Cl2 N4 O | -0.47406 | 0.010818 | 0.73021 |
| 2-Amino-3-methyl-1-butanol | 0.47165 | 0.011284 | 0.73021 |
| C35 H58 N2 O2 S2 | 0.46608 | 0.012425 | 0.73021 |
| 11-hydroxyandrost-4-ene-3_17-dione 8.046857 | -0.46417 | 0.01284 | 0.73021 |
| C12 H20 N2 O | -0.46295 | 0.013108 | 0.73021 |
| 882.7669@15.897112 | -0.45767 | 0.01433 | 0.73021 |
| 642.7101@8.700278 | -0.45703 | 0.014485 | 0.73021 |
| 998.6059@8.233334 | 0.45592 | 0.014755 | 0.73021 |
| C23 H49 N3 O S | 0.44729 | 0.01701 | 0.73021 |
| Jasmonic acid 6.6657147 | -0.44727 | 0.017013 | 0.73021 |
| 877.5794@13.109153 | 0.4472 | 0.017034 | 0.73021 |
| 445.7507@12.445499 | -0.44642 | 0.017251 | 0.73021 |
| C27 H27 N11 O8 | -0.44642 | 0.017251 | 0.73021 |
| C9 H19 N7 O | -0.44536 | 0.01755 | 0.73021 |
| C9 H22 N8 O | -0.44317 | 0.018181 | 0.73021 |
| C47 H72 O3 S2 | 0.44164 | 0.018632 | 0.73021 |
| 809.5469@12.846333 | 0.43858 | 0.019562 | 0.73021 |
| C12 H24 N2 O3 | -0.43824 | 0.01967 | 0.73021 |
| L-Tyrosine | -0.43545 | 0.020556 | 0.73021 |
| C16 H35 N5 O S | -0.43531 | 0.020598 | 0.73021 |
| trans-Dehydroandrosterone 9.5154 | -0.43442 | 0.020889 | 0.73021 |
| C18 H39 N9 O | -0.43173 | 0.021787 | 0.73021 |
| C14 H34 N10 O4 | 0.43154 | 0.021852 | 0.73021 |
| 1034.6356@11.147 | 0.43047 | 0.022216 | 0.73021 |
| C29 H51 N13 S2 | -0.42618 | 0.023737 | 0.73021 |
| 433.7505@6.662929 | -0.42475 | 0.02426 | 0.73021 |
| 1220.8862@13.607 | -0.42158 | 0.025456 | 0.73021 |
| C19 H45 Cl2 N9 | -0.42043 | 0.025901 | 0.73021 |
| C43 H47 N5 O4 | -0.42041 | 0.025909 | 0.73021 |
| 915.5582@7.3104777 | -0.41994 | 0.026094 | 0.73021 |
| 941.3513@12.901501 | -0.41974 | 0.026172 | 0.73021 |
| C32 H57 N O10 S | -0.41939 | 0.026309 | 0.73021 |
| 1193.319@13.169749 | 0.4189 | 0.026505 | 0.73021 |
| 789.2404@13.022819 | -0.4177 | 0.026986 | 0.73021 |
| C16 H38 N10 O6 | -0.41683 | 0.027341 | 0.73021 |
| C32 H47 N11 O4 | -0.41642 | 0.027508 | 0.73021 |
| 793.5179@11.174819 | -0.41613 | 0.027626 | 0.73021 |
| 1593.1213@13.165127 | 0.41608 | 0.027646 | 0.73021 |
| 1187.8757@13.586 | -0.41505 | 0.028073 | 0.73021 |
| C15 H35 N13 | -0.41474 | 0.028204 | 0.73021 |
| C22 H37 N9 O4 | -0.41303 | 0.028925 | 0.73021 |
| C29 H39 N7 | -0.41269 | 0.029071 | 0.73021 |
| 1196.8661@13.56 | 0.41269 | 0.029071 | 0.73021 |
| C18 H11 N7 O11 S2 | -0.41198 | 0.029379 | 0.73021 |
| 1239.8026@13.0825 | 0.41146 | 0.029601 | 0.73021 |
| 758.2253@13.449889 | -0.41045 | 0.030044 | 0.73021 |
| 887.7976@15.256616 | -0.40987 | 0.030301 | 0.73021 |
| C16 H36 N4 O2 | 0.40987 | 0.030301 | 0.73021 |
| 927.834@15.661571 | -0.40871 | 0.030818 | 0.73021 |
| 516.8406@10.884167 | -0.40825 | 0.031024 | 0.73021 |
| 923.2825@13.084251 | 0.40675 | 0.031708 | 0.73021 |
| C36 H4 O17 S | -0.40646 | 0.031842 | 0.73021 |
| C13 H9 N3 O | -0.40502 | 0.032512 | 0.73021 |
| C34 H65 N S2 | -0.40502 | 0.032512 | 0.73021 |
| C45 H63 N O2 | -0.40502 | 0.032512 | 0.73021 |
| 1071.321@13.397142 | -0.40499 | 0.032528 | 0.73021 |
| C26 H51 N13 O5 S | -0.40484 | 0.032596 | 0.73021 |
| 370.088@11.565685 | -0.40467 | 0.032677 | 0.73021 |
| C24 H42 N4 O3 | -0.4029 | 0.033519 | 0.73021 |
| C6 H6 S3 | -0.40196 | 0.033976 | 0.73021 |
| C11 H17 N5 O | -0.40183 | 0.034037 | 0.73021 |
| C23 H32 N2 O | -0.40148 | 0.034211 | 0.73021 |
| C35 H59 N3 O3 S3 | -0.39863 | 0.035627 | 0.73021 |
| C20 H40 N10 O3 | -0.3981 | 0.035893 | 0.73021 |
| C21 H28 O8 | 0.39755 | 0.036176 | 0.73021 |
| 951.3111@13.671 | 0.39736 | 0.036272 | 0.73021 |
| C19 H34 N4 O5 | -0.39732 | 0.036291 | 0.73021 |
| 780.4043@7.3505 | -0.39573 | 0.037115 | 0.73021 |
| C17 H3 N3 O9 S2 | -0.3941 | 0.037976 | 0.73021 |
| C38 H72 N2 O4 S4 | -0.39408 | 0.037984 | 0.73021 |
| C21 H36 Cl2 N6 O11 | -0.39333 | 0.038384 | 0.73021 |
| 1541.1049@13.111 | 0.39245 | 0.038857 | 0.73021 |
| C19 H33 N7 | 0.3914 | 0.039432 | 0.73021 |
| C17 H40 N4 O S3 | -0.39109 | 0.039604 | 0.73021 |
| C31 H56 N2 S2 | -0.39006 | 0.04017 | 0.73021 |
| 1262.3584@13.644333 | 0.38911 | 0.040699 | 0.73021 |
| C17 H46 N10 O8 S | -0.38899 | 0.04077 | 0.73021 |
| C15 H28 N4 O3 | -0.38847 | 0.041064 | 0.73021 |
| C16 H30 O5 | -0.38847 | 0.041064 | 0.73021 |
| L-Tryptophan | 0.38811 | 0.041265 | 0.73021 |
| C13 H25 N5 O2 S | -0.38685 | 0.041988 | 0.73021 |
| 942.5462@8.220201 | -0.38678 | 0.042027 | 0.73021 |
| C23 H10 N2 O12 S4 | -0.38583 | 0.042577 | 0.73021 |
| C14 H26 N8 O3 5.9993935 | -0.38565 | 0.042684 | 0.73021 |
| C27 H44 O6 | 0.38557 | 0.042729 | 0.73021 |
| 629.7112@8.70054 | -0.38481 | 0.043178 | 0.73021 |
| 752.1111@8.70125 | -0.38467 | 0.043259 | 0.73021 |
| C15 H40 N8 O4 S | -0.38462 | 0.043287 | 0.73021 |
| C21 H34 N4 | 0.38455 | 0.043328 | 0.73021 |
| C4 H8 | -0.38418 | 0.04355 | 0.73021 |
| C34 H47 N7 O S | -0.38406 | 0.043621 | 0.73021 |
| C34 H73 N7 | -0.38392 | 0.0437 | 0.73021 |
| C38 H75 N5 | -0.38367 | 0.043852 | 0.73021 |
| 774.4155@7.349499 | 0.38314 | 0.044166 | 0.73021 |
| C17 H21 Cl N2 S2 | 0.383 | 0.044251 | 0.73021 |
| 604.685@8.121639 | -0.38284 | 0.044345 | 0.73021 |
| C36 H36 Cl2 O8 | -0.38236 | 0.044637 | 0.73021 |
| 476.7839@8.7095995 | -0.3822 | 0.044732 | 0.73021 |
| C14 H30 N4 O | 0.381 | 0.045467 | 0.73021 |
| C11 H19 N O | -0.38098 | 0.045476 | 0.73021 |
| C19 H19 N5 O12 S3 | -0.38069 | 0.045656 | 0.73021 |
| C32 H16 N4 O5 S | -0.38065 | 0.04568 | 0.73021 |
| 450.7989@10.762 | -0.38042 | 0.045825 | 0.73021 |
| C15 H39 Cl N16 O4 | -0.37925 | 0.046549 | 0.73021 |
| 579.6746@8.4978695 | -0.37874 | 0.046868 | 0.73021 |
| C38 H30 Cl N3 O4 | -0.37847 | 0.047041 | 0.73021 |
| C22 H47 N3 O6 S | -0.37754 | 0.047629 | 0.73021 |
| 837.7845@15.485 | -0.37712 | 0.047894 | 0.73021 |
| 751.7761@8.6996155 | -0.37693 | 0.048015 | 0.73021 |
| 79.9407@0.38566667 | -0.37677 | 0.048121 | 0.73021 |
| Chrysin | -0.37655 | 0.048265 | 0.73021 |
| 1127.3153@14.397584 | -0.37495 | 0.049302 | 0.73021 |
| C6 H17 N7 O3 | 0.37481 | 0.04939 | 0.73021 |
| 777.5618@13.068742 | -0.3742 | 0.049796 | 0.73021 |
| 875.6179@13.431315 | 0.37404 | 0.0499 | 0.73021 |

P value after Spearman correlation analyses. FDR: False-discovery corrected p value after Benjamini-Hochberg correction. * ions presented are preliminary characterized by potential elemental formulae or, when it is not possible, characterized by m/z and retention time (in minutes) separated by the @ symbol
