# Supplementary material for: Differential metabolic profiles associated to movement behaviour of stream-resident brown trout (Salmo trutta)
Source: PLoS One. 2017 Jul 27;12(7):e0181697. doi: 10.1371/journal.pone.0181697 (PMC5531495; doi:10.1371/journal.pone.0181697)
Supplement: S3 Table — (DOCX) [file pone.0181697.s003.docx]

**S3 Table. Molecules correlating with home range.**

| **Molecule*** | **correlation** | **p-value** | **FDR** |
| --- | --- | --- | --- |
| C32 H57 N7 O3 S | -0.91495 | 9.545e-12 | 1.6413e-08 |
| 873.7847@15.069066 | -0.89973 | 7.4223e-11 | 7.0157e-08 |
| 1275.3534@15.064867 | -0.89863 | 8.5043e-11 | 7.0157e-08 |
| 795.5323@10.679536 | -0.89713 | 1.02e-10 | 7.0157e-08 |
| C26 H49 N3 O4 | -0.89388 | 1.5002e-10 | 8.4327e-08 |
| C45 H89 N O6 | -0.88852 | 2.7595e-10 | 8.4327e-08 |
| 817.7275@14.412071 | -0.88794 | 2.9396e-10 | 8.4327e-08 |
| 843.753@14.486929 | -0.8877 | 3.0207e-10 | 8.4327e-08 |
| C27 H53 N17 O3 | -0.88723 | 3.179e-10 | 8.4327e-08 |
| 863.7946@15.591927 | -0.88661 | 3.4009e-10 | 8.4327e-08 |
| 809.7409@15.003928 | -0.8866 | 3.4058e-10 | 8.4327e-08 |
| 877.8214@15.900642 | -0.88624 | 3.5411e-10 | 8.4327e-08 |
| 835.767@15.082643 | -0.8861 | 3.5961e-10 | 8.4327e-08 |
| 903.8304@16.012714 | -0.88589 | 3.6781e-10 | 8.4327e-08 |
| C32 H65 N7 O4 S | -0.88255 | 5.2497e-10 | 1.1284e-07 |
| 339.7606@7.3483334 | 0.87926 | 7.3736e-10 | 1.4916e-07 |
| C26 H45 N7 S | -0.87348 | 1.3085e-09 | 2.5e-07 |
| C22 H47 N9 O2 S | -0.87282 | 1.3945e-09 | 2.5241e-07 |
| C27 H53 N3 O5 S | -0.8709 | 1.6754e-09 | 2.8808e-07 |
| 2-Octenoic acid | -0.86554 | 2.7539e-09 | 4.5099e-07 |
| C25 H42 O S | -0.86148 | 3.9594e-09 | 6.1893e-07 |
| Retinaldehyde | -0.85946 | 4.7221e-09 | 7.0606e-07 |
| 851.7961@15.834309 | -0.85231 | 8.6241e-09 | 1.2358e-06 |
| 791.6999@14.344232 | -0.84939 | 1.0934e-08 | 1.4881e-06 |
| C29 H57 N3 O6 S | -0.8489 | 1.1374e-08 | 1.4881e-06 |
| C21 H53 N9 O3 S | -0.84856 | 1.1683e-08 | 1.4881e-06 |
| 859.7633@14.869666 | -0.84799 | 1.2234e-08 | 1.4945e-06 |
| C12 H26 N4 O | 0.84761 | 1.2602e-08 | 1.4945e-06 |
| Diethyl Oxalpropionate | 0.84334 | 1.7595e-08 | 1.9828e-06 |
| C34 H63 N7 O8 | 0.84314 | 1.7874e-08 | 1.9828e-06 |
| C25 H45 N3 O3 | -0.8371 | 2.817e-08 | 3.0274e-06 |
| 839.558@10.722678 | -0.83575 | 3.1103e-08 | 3.2414e-06 |
| 901.8259@15.522461 | -0.8344 | 3.4314e-08 | 3.4708e-06 |
| 854.7248@15.334614 | -0.83258 | 3.9133e-08 | 3.7498e-06 |
| 1247.3746@14.438691 | -0.83222 | 4.0157e-08 | 3.7498e-06 |
| 895.7734@14.630286 | -0.83115 | 4.3319e-08 | 3.7498e-06 |
| C20 H38 N2 O2 S | 0.83107 | 4.3592e-08 | 3.7498e-06 |
| 767.6967@14.508359 | -0.83106 | 4.3608e-08 | 3.7498e-06 |
| 819.7323@14.622213 | -0.83106 | 4.3615e-08 | 3.7498e-06 |
| 869.7499@14.567857 | -0.83064 | 4.4944e-08 | 3.7698e-06 |
| C26 H51 N17 O2 | -0.82624 | 6.108e-08 | 4.9223e-06 |
| 795.735@14.832078 | -0.82613 | 6.1546e-08 | 4.9223e-06 |
| 833.7491@14.782769 | -0.82533 | 6.5023e-08 | 5.0822e-06 |
| 871.7682@14.797922 | -0.82439 | 6.9301e-08 | 5.2198e-06 |
| 807.7282@14.708076 | -0.82428 | 6.982e-08 | 5.2198e-06 |
| 1201.3309@14.693768 | -0.82269 | 7.7736e-08 | 5.688e-06 |
| 861.7883@15.169001 | -0.82216 | 8.0546e-08 | 5.7708e-06 |
| C37 H61 N11 O S | -0.81927 | 9.7654e-08 | 6.8016e-06 |
| 1127.3153@14.397584 | -0.819 | 9.9369e-08 | 6.8016e-06 |
| C24 H51 N11 O2 | 0.81877 | 1.0087e-07 | 6.8016e-06 |
| C17 H45 N9 O S | -0.81746 | 1.099e-07 | 7.2679e-06 |
| 885.7812@14.963859 | -0.81555 | 1.2444e-07 | 8.0747e-06 |
| 847.7654@14.9754 | -0.81519 | 1.2733e-07 | 8.1093e-06 |
| C26 H51 N13 O5 S | -0.81377 | 1.3944e-07 | 8.719e-06 |
| 1193.6969@7.3845015 | -0.81005 | 1.7633e-07 | 1.0829e-05 |
| C19 H49 N23 | -0.80886 | 1.8985e-07 | 1.1454e-05 |
| C16 H27 N3 S | -0.80768 | 2.0413e-07 | 1.1944e-05 |
| C36 H69 N O5 S | -0.80762 | 2.0492e-07 | 1.1944e-05 |
| C23 H51 N13 O5 | -0.80217 | 2.8504e-07 | 1.6337e-05 |
| 821.7491@14.898713 | -0.80065 | 3.1195e-07 | 1.7587e-05 |
| C34 H63 N11 O6 | -0.79936 | 3.3658e-07 | 1.8669e-05 |
| C18 H5 N5 O13 S3 | -0.79833 | 3.575e-07 | 1.9515e-05 |
| C15 H3 N3 O11 S4 | -0.79774 | 3.7e-07 | 1.9882e-05 |
| 462.8253@11.058181 | -0.79697 | 3.869e-07 | 2.047e-05 |
| 826.7079@14.901856 | -0.79663 | 3.9469e-07 | 2.0566e-05 |
| 897.7802@14.869667 | -0.79296 | 4.8682e-07 | 2.4988e-05 |
| 893.7575@14.4455 | -0.78837 | 6.2959e-07 | 3.184e-05 |
| 1053.2913@14.153635 | -0.78692 | 6.8176e-07 | 3.3979e-05 |
| 849.7833@15.328334 | -0.78395 | 8.0149e-07 | 3.9376e-05 |
| C12 H22 N2 O2 | 0.78286 | 8.501e-07 | 4.1176e-05 |
| C24 H53 N13 S | -0.77875 | 1.0573e-06 | 5.0501e-05 |
| 845.751@14.699154 | -0.77776 | 1.1138e-06 | 5.2473e-05 |
| C20 H7 N7 O9 S4 | -0.77463 | 1.3105e-06 | 6.0904e-05 |
| 440.8134@11.025538 | -0.77099 | 1.578e-06 | 7.2355e-05 |
| 759.4928@12.662937 | -0.769 | 1.7446e-06 | 7.8844e-05 |
| 889.8143@15.666751 | -0.76876 | 1.7653e-06 | 7.8844e-05 |
| 839.4983@8.987667 | -0.76826 | 1.8101e-06 | 7.9805e-05 |
| C20 H22 O | -0.76712 | 1.9156e-06 | 8.3389e-05 |
| C20 H51 N11 O8 S | -0.76316 | 2.3278e-06 | 0.00010007 |
| C22 H38 N6 O2 | -0.76249 | 2.4039e-06 | 0.00010206 |
| 775.2294@12.662143 | -0.76122 | 2.5569e-06 | 0.00010723 |
| C41 H30 Cl N9 O2 | -0.75977 | 2.7408e-06 | 0.00011356 |
| C36 H74 N4 O5 S | -0.75937 | 2.7943e-06 | 0.0001144 |
| 1173.356@14.2078 | -0.75877 | 2.8747e-06 | 0.00011456 |
| C21 H3 N O15 S2 | -0.75869 | 2.8866e-06 | 0.00011456 |
| C27 H61 N17 O4 | -0.75822 | 2.9522e-06 | 0.00011456 |
| 550.8822@11.171499 | -0.7581 | 2.9691e-06 | 0.00011456 |
| C47 H85 N O S | -0.75808 | 2.9719e-06 | 0.00011456 |
| 484.8405@11.089098 | -0.75789 | 2.9982e-06 | 0.00011456 |
| 800.5507@12.606823 | -0.75447 | 3.5224e-06 | 0.00013312 |
| C35 H34 N12 S3 | -0.75416 | 3.5738e-06 | 0.00013359 |
| C20 H43 N9 O S | -0.74578 | 5.2439e-06 | 0.00019391 |
| C20 H47 N13 O6 S | -0.74198 | 6.2079e-06 | 0.00022712 |
| 793.7203@14.5632305 | -0.73884 | 7.1236e-06 | 0.000256 |
| C23 H53 N5 S2 | -0.73877 | 7.1464e-06 | 0.000256 |
| C26 H46 N4 O2 | -0.73721 | 7.6464e-06 | 0.00027109 |
| C28 H62 N2 O2 | 0.73603 | 8.0452e-06 | 0.00027992 |
| C28 H55 N3 O5 S | -0.73599 | 8.0582e-06 | 0.00027992 |
| 899.7987@15.147118 | -0.7356 | 8.1943e-06 | 0.0002818 |
| C29 H57 N5 O3 S3 | -0.73418 | 8.7063e-06 | 0.00029644 |
| C46 H91 N O S2 | -0.73371 | 8.8829e-06 | 0.00029949 |
| C31 H32 N12 O2 | 0.73308 | 9.1255e-06 | 0.00030469 |
| 802.5418@11.946736 | -0.73123 | 9.8663e-06 | 0.00032625 |
| 887.7976@15.256616 | -0.72956 | 1.0584e-05 | 0.00034664 |
| 401.7547@12.811786 | -0.72902 | 1.0822e-05 | 0.0003511 |
| C29 H62 Cl N3 S | -0.72726 | 1.1641e-05 | 0.00037416 |
| 880.7552@15.407144 | -0.72559 | 1.2474e-05 | 0.00039719 |
| C31 H63 N7 O9 | -0.71855 | 1.6579e-05 | 0.00052309 |
| 779.4475@8.0654 | -0.71665 | 1.788e-05 | 0.00055899 |
| 873.6415@12.256 | -0.71603 | 1.8319e-05 | 0.00056756 |
| 770.4767@10.9727335 | -0.713 | 2.0632e-05 | 0.00063352 |
| C36 H71 N3 O12 | -0.71159 | 2.1797e-05 | 0.00066335 |
| 823.7642@15.267615 | -0.70965 | 2.3484e-05 | 0.00070843 |
| 1349.3726@15.545363 | -0.70833 | 2.4705e-05 | 0.00073879 |
| 783.5009@12.772285 | -0.70298 | 3.0242e-05 | 0.00089658 |
| 911.6166@11.262134 | -0.70127 | 3.2232e-05 | 0.00094741 |
| C41 H39 N3 S4 | -0.70098 | 3.2575e-05 | 0.00094938 |
| 683.536@12.4502 | -0.70016 | 3.3586e-05 | 0.00097061 |
| C33 H16 O9 S2 | 0.69858 | 3.5589e-05 | 0.0010199 |
| C39 H34 Cl N5 O S | -0.69786 | 3.6539e-05 | 0.0010385 |
| 867.7459@14.381222 | -0.69451 | 4.128e-05 | 0.0011545 |
| C36 H51 N3 | -0.6945 | 4.129e-05 | 0.0011545 |
| C7 H12 O | 0.69333 | 4.3079e-05 | 0.0011948 |
| C30 H30 Cl N O4 S | -0.69262 | 4.419e-05 | 0.0012158 |
| 787.5161@12.884998 | -0.69058 | 4.7537e-05 | 0.0012975 |
| 452.8335@8.188178 | -0.68899 | 5.0294e-05 | 0.0013619 |
| 761.5026@12.792551 | -0.68413 | 5.9615e-05 | 0.0015943 |
| C18 H39 N9 S | -0.68404 | 5.9805e-05 | 0.0015943 |
| C38 H72 N2 O4 S4 | -0.68311 | 6.1764e-05 | 0.0016339 |
| C13 H31 N5 | 0.67971 | 6.9431e-05 | 0.0018227 |
| 1321.3921@14.7263 | -0.6791 | 7.088e-05 | 0.0018466 |
| C30 H35 Cl N6 O6 | -0.6761 | 7.8456e-05 | 0.0020131 |
| C29 H65 N7 O3 S4 | -0.67598 | 7.8776e-05 | 0.0020131 |
| 759.5356@11.152778 | -0.67589 | 7.9025e-05 | 0.0020131 |
| C34 H53 N O | -0.67537 | 8.0408e-05 | 0.0020333 |
| 875.8096@15.408001 | -0.67335 | 8.602e-05 | 0.0021593 |
| 814.5039@11.006277 | -0.67257 | 8.828e-05 | 0.0021988 |
| C36 H4 N4 O7 S3 | -0.67237 | 8.8874e-05 | 0.0021988 |
| C21 H2 O18 S2 | -0.67017 | 9.5579e-05 | 0.0023478 |
| C40 H76 Cl N O4 | -0.66934 | 9.823e-05 | 0.0023958 |
| 763.662@14.115499 | -0.66499 | 0.00011315 | 0.0027404 |
| 1233.355@13.931 | -0.66307 | 0.00012037 | 0.0028948 |
| 778.228@12.66409 | -0.66172 | 0.00012567 | 0.0030013 |
| C12 H14 N2 | -0.66149 | 0.00012663 | 0.0030033 |
| C22 H44 O2 S | 0.65959 | 0.0001345 | 0.0031682 |
| C40 H61 N7 S | -0.65602 | 0.00015049 | 0.0035207 |
| C32 H30 N2 S3 | -0.6549 | 0.00015584 | 0.0036212 |
| C27 H5 N O23 | -0.65457 | 0.00015743 | 0.0036337 |
| C5 H8 O3 S | 0.654 | 0.00016024 | 0.0036738 |
| C13 H29 N5 | -0.65206 | 0.00017016 | 0.0038754 |
| C13 H N O15 S3 | -0.65027 | 0.00017978 | 0.0040674 |
| D-Galactose | -0.64918 | 0.0001859 | 0.0041784 |
| C20 H40 N10 O3 | -0.64887 | 0.00018761 | 0.0041896 |
| C28 H33 Cl N6 O6 | -0.63988 | 0.00024571 | 0.0054516 |
| 955.6445@11.286356 | -0.63905 | 0.00025174 | 0.0055495 |
| C35 H69 N5 S | -0.63601 | 0.0002752 | 0.0060281 |
| ?-Caprolactam | 0.63494 | 0.00028385 | 0.0061782 |
| 792.094@9.513882 | -0.63448 | 0.00028774 | 0.0062235 |
| C16 H36 N16 | -0.63356 | 0.00029542 | 0.0063497 |
| 1073.7112@11.180459 | -0.6286 | 0.00034056 | 0.0072743 |
| 995.6983@11.11352 | -0.62781 | 0.00034824 | 0.0073927 |
| Oleamide | -0.62482 | 0.00037886 | 0.0079933 |
| 1029.684@11.151361 | -0.62417 | 0.00038574 | 0.008042 |
| 942.5462@8.220201 | -0.62407 | 0.00038682 | 0.008042 |
| 979.2757@13.947445 | -0.62362 | 0.00039176 | 0.008042 |
| 1307.377@14.1071415 | -0.62357 | 0.00039233 | 0.008042 |
| O-Benzyl-L-Tyrosine | 0.62352 | 0.00039286 | 0.008042 |
| C21 H13 N9 O14 S2 | -0.62295 | 0.00039915 | 0.0080826 |
| C38 H69 N7 O S | -0.62291 | 0.00039955 | 0.0080826 |
| 1161.7672@11.232572 | -0.62188 | 0.00041122 | 0.00827 |
| Methyl linolenate | -0.62059 | 0.00042615 | 0.0085206 |
| 951.6717@11.084481 | -0.61998 | 0.00043334 | 0.0086141 |
| C18 H4 N4 O17 S2 | -0.61954 | 0.00043862 | 0.008669 |
| C40 H82 N4 O S2 | -0.61836 | 0.00045307 | 0.0089035 |
| C23 H10 N2 O12 S4 | -0.61801 | 0.00045748 | 0.008939 |
| 749.4882@11.144919 | -0.61729 | 0.00046659 | 0.0090655 |
| Estrone | -0.61681 | 0.00047272 | 0.0091331 |
| C25 H2 O14 S2 | -0.61456 | 0.00050252 | 0.0096546 |
| C34 H67 N5 O S2 | -0.61422 | 0.00050704 | 0.0096873 |
| 985.6564@11.124041 | -0.61396 | 0.00051073 | 0.009695 |
| 841.7254@14.314665 | -0.61379 | 0.00051308 | 0.009695 |
| 941.6291@11.094922 | -0.61229 | 0.00053423 | 0.010039 |
| 837.5427@12.905668 | -0.61022 | 0.0005646 | 0.010552 |
| C19 H46 N14 O5 | -0.60796 | 0.00059956 | 0.011108 |
| C36 H52 N4 S | -0.60788 | 0.00060078 | 0.011108 |
| 752.4292@7.359958 | -0.60729 | 0.00061028 | 0.011223 |
| C38 H69 N7 O5 S | -0.60656 | 0.00062208 | 0.01138 |
| C17 H38 N8 O | -0.6059 | 0.00063302 | 0.011518 |
| 823.5626@11.200417 | -0.60484 | 0.00065091 | 0.011781 |
| C17 H39 N15 O3 | -0.60335 | 0.00067675 | 0.012185 |
| 11?-hydroxyandrost-4-ene-3_17-dione + 8.821813 | -0.60291 | 0.00068458 | 0.012232 |
| C26 H42 N4 O4 | 0.60278 | 0.0006868 | 0.012232 |
| 545.8607@11.178124 | -0.6026 | 0.00069 | 0.012232 |
| 444.7809@7.1436253 | -0.60189 | 0.00070293 | 0.012397 |
| C37 H62 N2 O3 S2 | 0.60166 | 0.0007071 | 0.012407 |
| 867.59@11.232364 | -0.60019 | 0.00073458 | 0.012823 |
| C21 H35 N3 | -0.59974 | 0.00074311 | 0.012907 |
| C34 H65 N3 O2 | -0.59927 | 0.00075203 | 0.012965 |
| C28 H N O11 S3 | -0.59917 | 0.00075399 | 0.012965 |
| 501.8338@11.125001 | -0.59894 | 0.00075843 | 0.012976 |
| C18 H41 N9 O6 | -0.59841 | 0.0007688 | 0.013089 |
| C16 H46 N10 O8 | -0.5979 | 0.00077905 | 0.013198 |
| C23 H12 O17 S3 | -0.59602 | 0.00081725 | 0.013777 |
| 530.4004@12.789599 | -0.59504 | 0.00083792 | 0.014057 |
| C27 H29 Cl N2 O10 S3 | -0.5947 | 0.00084517 | 0.014109 |
| 1176.35@14.205 | -0.59331 | 0.00087537 | 0.014543 |
| N-Oleoyl-D-erythro-Sphingosine (C18:1 Ceramide) | -0.59246 | 0.00089427 | 0.014738 |
| 778.899@9.2304 | 0.5924 | 0.00089565 | 0.014738 |
| C16 H34 N2 O | 0.5917 | 0.00091165 | 0.014929 |
| 465.5191@11.711858 | -0.59059 | 0.00093725 | 0.015276 |
| C27 H47 N3 O | -0.58972 | 0.00095775 | 0.015536 |
| 1083.7544@11.167809 | -0.58899 | 0.00097531 | 0.015747 |
| C16 H20 N2 O18 | -0.58828 | 0.00099269 | 0.015953 |
| 892.0344@8.57423 | -0.58693 | 0.0010264 | 0.016418 |
| C16 H34 N4 O3 | -0.58648 | 0.0010379 | 0.016525 |
| 781.7139@14.631499 | -0.58488 | 0.0010797 | 0.017111 |
| 572.9946@8.445429 | -0.58388 | 0.0011064 | 0.017454 |
| 655.928@11.293688 | -0.58346 | 0.0011178 | 0.017553 |
| C22 H40 Cl N O6 | -0.58271 | 0.0011386 | 0.017798 |
| C27 H N O5 S5 | -0.58181 | 0.0011637 | 0.018109 |
| C30 H46 N14 S | -0.58096 | 0.0011881 | 0.018405 |
| C18 H30 N8 O4 | 0.57956 | 0.0012288 | 0.018899 |
| C29 H57 N O10 | -0.57949 | 0.001231 | 0.018899 |
| C12 H37 N15 O S2 | -0.57714 | 0.0013025 | 0.019908 |
| 775.5259@12.948999 | -0.57624 | 0.0013308 | 0.020251 |
| 637.8385@7.539286 | -0.57486 | 0.0013753 | 0.020751 |
| C16 H22 N4 O12 S4 | -0.57472 | 0.0013801 | 0.020751 |
| C37 H58 Cl N O2 | -0.57467 | 0.0013818 | 0.020751 |
| C18 H36 N16 O3 | -0.57426 | 0.0013953 | 0.020835 |
| 5?-dihydroprogesterone | -0.57413 | 0.0013995 | 0.020835 |
| 16b-Hydroxyestradiol | -0.57267 | 0.0014485 | 0.021472 |
| 619.697@8.183667 | -0.57033 | 0.0015306 | 0.022591 |
| 11?-hydroxyandrost-4-ene-3_17-dione + 8.046857 | -0.56738 | 0.0016398 | 0.024099 |
| 812.6812@13.836545 | -0.56469 | 0.0017451 | 0.025537 |
| C38 H69 N S2 | -0.56429 | 0.0017611 | 0.025663 |
| 1099.3359@14.006 | -0.56403 | 0.0017716 | 0.025707 |
| C24 H48 S | -0.56327 | 0.0018026 | 0.025983 |
| 894.6927@12.621857 | -0.5632 | 0.0018058 | 0.025983 |
| 1237.7275@7.541429 | -0.56263 | 0.0018294 | 0.026213 |
| 876.5087@8.011856 | -0.56191 | 0.0018598 | 0.026464 |
| C36 H65 N O3 S2 | -0.56177 | 0.0018658 | 0.026464 |
| 781.5749@13.304871 | -0.56167 | 0.0018699 | 0.026464 |
| 421.778@12.963562 | -0.56148 | 0.0018781 | 0.02647 |
| 766.1192@8.738857 | -0.56052 | 0.0019196 | 0.026945 |
| C31 H48 O S | -0.55916 | 0.0019796 | 0.027615 |
| C17 H31 N5 O | -0.55908 | 0.0019834 | 0.027615 |
| 882.7669@15.897112 | -0.557 | 0.0020784 | 0.02882 |
| 663.7236@8.372822 | -0.55614 | 0.0021189 | 0.029264 |
| 3-Methylglutaric acid + 1.4608947 | 0.55517 | 0.0021656 | 0.02979 |
| C10 H22 N2 O4 | 0.55471 | 0.002188 | 0.029978 |
| 1423.3965@16.184332 | -0.55421 | 0.0022126 | 0.030196 |
| TOFA | 0.55371 | 0.0022371 | 0.030409 |
| 426.41@12.643001 | 0.55316 | 0.0022648 | 0.030664 |
| 891.532@8.576555 | -0.55207 | 0.0023204 | 0.031293 |
| C43 H57 N11 O | -0.55183 | 0.0023323 | 0.031331 |
| 971.967@13.090571 | -0.55123 | 0.0023636 | 0.031562 |
| 839.711@14.129201 | -0.55115 | 0.0023679 | 0.031562 |
| 585.6852@8.50307 | -0.54986 | 0.002436 | 0.032236 |
| C12 Cl3 N3 O2 S4 | -0.54984 | 0.0024372 | 0.032236 |
| C31 H32 N12 O2 + 5.997273 | 0.54949 | 0.0024562 | 0.032363 |
| C28 H49 N17 O2 | -0.54795 | 0.0025402 | 0.033343 |
| C19 H47 N13 O6 S | -0.54728 | 0.0025777 | 0.033639 |
| C23 H53 N23 O S | -0.54711 | 0.0025876 | 0.033639 |
| C14 H32 N2 O9 | -0.54702 | 0.0025921 | 0.033639 |
| 763.5218@12.949665 | -0.54661 | 0.0026159 | 0.033698 |
| C19 H44 Cl N13 O2 | -0.54656 | 0.0026183 | 0.033698 |
| C35 H16 N8 O2 S | -0.54643 | 0.0026261 | 0.033698 |
| C22 H30 N2 | -0.54456 | 0.0027348 | 0.034962 |
| 1081.3381@14.1505 | -0.54327 | 0.0028117 | 0.035813 |
| 837.7012@13.958501 | -0.5428 | 0.0028404 | 0.036044 |
| C30 H65 N3 O S5 | -0.54147 | 0.002923 | 0.036847 |
| C12 H28 N2 O3 S2 | 0.54143 | 0.0029251 | 0.036847 |
| C42 H69 N S2 | -0.5407 | 0.0029716 | 0.037297 |
| 1204.3279@14.696143 | -0.54042 | 0.0029893 | 0.037382 |
| C25 H49 N13 O5 | -0.53796 | 0.0031496 | 0.039245 |
| C39 H76 N2 O S3 | -0.5368 | 0.0032284 | 0.040081 |
| 616.4222@11.239714 | -0.53383 | 0.0034361 | 0.042506 |
| C24 H44 N2 O7 | -0.53362 | 0.0034512 | 0.04254 |
| 614.7021@8.635143 | -0.53271 | 0.0035181 | 0.043165 |
| 907.6445@11.052731 | -0.53258 | 0.003527 | 0.043165 |
| C22 H29 N O | 0.53188 | 0.0035794 | 0.04365 |
| 978.6101@5.89425 | 0.53141 | 0.0036143 | 0.043921 |
| C29 H65 N5 O10 S2 | -0.53122 | 0.0036289 | 0.043941 |
| 924.3864@12.920875 | -0.53105 | 0.0036415 | 0.043941 |
| C22 H20 O | 0.5292 | 0.0037833 | 0.045493 |
| 590.68@8.054 | -0.52822 | 0.0038613 | 0.046268 |
| 837.5439@11.203501 | -0.52775 | 0.0038984 | 0.046551 |
| C27 H10 N2 O7 S5 | -0.52679 | 0.0039759 | 0.047312 |
| 950.3994@12.967875 | -0.5266 | 0.0039913 | 0.047331 |
| C21 H39 N3 S | 0.52576 | 0.0040605 | 0.047882 |
| C36 H67 N S2 | -0.5257 | 0.0040656 | 0.047882 |
| C38 H30 Cl N3 O4 | -0.52491 | 0.0041318 | 0.048256 |
| C39 H67 N5 O S | -0.52483 | 0.0041385 | 0.048256 |
| C23 H38 N2 O2 S | 0.52459 | 0.0041586 | 0.048256 |
| 792.5202@10.995273 | 0.52452 | 0.0041652 | 0.048256 |
| C27 H38 N12 O | -0.52449 | 0.0041675 | 0.048256 |
| C38 H57 N3 O S | -0.52324 | 0.0042744 | 0.049191 |
| C33 H72 N10 O3 S | -0.52321 | 0.0042769 | 0.049191 |
| C41 H85 N3 O S | -0.52276 | 0.0043166 | 0.04923 |
| 813.5323@12.954801 | -0.52268 | 0.0043231 | 0.04923 |
| C19 O25 | -0.52268 | 0.0043232 | 0.04923 |
| 1228.7924@13.084 | -0.52235 | 0.0043521 | 0.049395 |
| C36 H63 N7 O4 | -0.52063 | 0.0045059 | 0.050944 |
| 905.8443@16.648859 | -0.5205 | 0.0045182 | 0.050944 |
| C37 H65 N O11 | -0.52024 | 0.0045417 | 0.051042 |
| 787.6168@13.894537 | -0.52003 | 0.0045606 | 0.051087 |
| C17 H40 N4 O S3 | -0.51945 | 0.0046141 | 0.051355 |
| C13 H14 N4 O10 S4 | -0.51945 | 0.0046144 | 0.051355 |
| C21 H15 N9 O14 S | -0.51922 | 0.0046357 | 0.051427 |
| C23 H17 N3 O4 | 0.51858 | 0.0046953 | 0.05192 |
| C23 H35 N3 O | -0.51838 | 0.0047141 | 0.051938 |
| C21 H49 N13 O9 | -0.51824 | 0.0047272 | 0.051938 |
| C39 H72 N4 O6 | -0.51696 | 0.0048498 | 0.053057 |
| C39 H71 N5 O6 | -0.51685 | 0.0048598 | 0.053057 |
| C31 H26 N6 O9 S3 | -0.51645 | 0.0048986 | 0.053286 |
| C21 H34 N2 O S | -0.51632 | 0.0049118 | 0.053286 |
| 853.5761@11.03152 | -0.51579 | 0.0049639 | 0.053614 |
| 780.7952@8.785429 | -0.51569 | 0.0049732 | 0.053614 |
| 751.5314@13.006952 | -0.51445 | 0.0050966 | 0.054341 |
| C20 H23 N9 O15 S2 | -0.51439 | 0.0051027 | 0.054341 |
| C32 H61 N13 O2 S | -0.51436 | 0.0051058 | 0.054341 |
| 803.4769@9.176801 | -0.5143 | 0.0051122 | 0.054341 |
| C16 H44 N12 O3 S | -0.51415 | 0.0051272 | 0.054341 |
| C27 H61 N5 O9 S2 | -0.51407 | 0.0051354 | 0.054341 |
| 568.5846@8.71962 | -0.51319 | 0.0052253 | 0.055122 |
| C9 H22 N4 O S | -0.51259 | 0.0052878 | 0.055529 |
| C26 H58 Cl N5 S | -0.51251 | 0.0052962 | 0.055529 |
| 319.1978@10.149 | 0.51223 | 0.0053246 | 0.055657 |
| C15 H37 N5 O3 | -0.51139 | 0.0054139 | 0.056419 |
| 819.5897@10.986616 | -0.51078 | 0.0054783 | 0.056845 |
| 213.2398@7.653928 | 0.51069 | 0.0054878 | 0.056845 |
| C43 H62 N6 S2 | -0.51001 | 0.0055617 | 0.057438 |
| 799.5168@13.008887 | -0.50923 | 0.0056467 | 0.058116 |
| C20 H2 N2 O16 S2 | -0.5091 | 0.0056612 | 0.058116 |
| C40 H61 N3 O2 S | -0.50877 | 0.0056971 | 0.058304 |
| 811.0728@9.512667 | 0.50862 | 0.0057134 | 0.058304 |
| C20 H4 N2 O11 S5 | -0.50803 | 0.0057793 | 0.058732 |
| 1557.1014@13.1539 | 0.50794 | 0.0057895 | 0.058732 |
| C34 H72 N4 O | -0.50655 | 0.0059479 | 0.060162 |
| 86.0699@0.9105 | 0.50624 | 0.0059832 | 0.060341 |
| C14 H40 N12 O2 S | -0.506 | 0.0060114 | 0.060448 |
| C36 H35 N O7 S3 | -0.50521 | 0.0061029 | 0.061189 |
| 479.8207@11.096462 | -0.50434 | 0.0062062 | 0.061983 |
| C35 H51 N7 O3 | -0.50424 | 0.0062181 | 0.061983 |
| C29 H40 Cl2 N2 O10 | -0.50395 | 0.0062529 | 0.062149 |
| C25 H49 N O2 S2 | 0.50354 | 0.0063013 | 0.06245 |
| Taurocholic acid | -0.50317 | 0.0063461 | 0.062713 |
| C19 H48 N14 O6 S | -0.50244 | 0.006436 | 0.063419 |
| C27 H51 N17 O3 S | -0.50226 | 0.0064571 | 0.063446 |
| C31 H48 O | -0.50196 | 0.0064952 | 0.063602 |
| 824.5602@7.542 | -0.50184 | 0.00651 | 0.063602 |
| C24 H49 N21 O5 | -0.50168 | 0.0065298 | 0.063615 |
| 1250.3688@14.438286 | -0.50146 | 0.0065562 | 0.063692 |
| 811.5317@12.852917 | -0.5011 | 0.0066015 | 0.063951 |
| Methyl linolenate + 12.6101675 | -0.50075 | 0.0066453 | 0.064092 |
| Retinoic acid | -0.50069 | 0.0066534 | 0.064092 |
| Cortexolone | -0.50046 | 0.006683 | 0.0641 |
| 747.9381@9.016625 | -0.50033 | 0.0066984 | 0.0641 |
| 786.1222@8.619 | -0.50024 | 0.0067101 | 0.0641 |
| C43 H92 N6 | -0.49936 | 0.0068234 | 0.064947 |
| 297.2616@11.304143 | -0.49926 | 0.0068365 | 0.064947 |
| C37 H57 N3 | -0.49837 | 0.0069511 | 0.065853 |
| C18 H46 N22 S2 | -0.49799 | 0.0070012 | 0.066146 |
| C47 H72 O3 S2 | 0.49731 | 0.0070914 | 0.0666 |
| C33 H47 N7 O2 | -0.49703 | 0.0071287 | 0.0666 |
| C14 H39 N11 O6 | -0.49702 | 0.0071308 | 0.0666 |
| 927.834@15.661571 | -0.49692 | 0.0071434 | 0.0666 |
| 426.7788@11.035131 | -0.4969 | 0.0071461 | 0.0666 |
| C12 H19 N O2 | 0.49593 | 0.0072773 | 0.067464 |
| 982.2708@13.945287 | -0.49593 | 0.007278 | 0.067464 |
| C15 H28 N4 | -0.49535 | 0.0073569 | 0.068011 |
| C20 H42 N4 O3 S | -0.49505 | 0.0073988 | 0.068215 |
| C36 H73 Cl N4 O2 S | -0.49479 | 0.0074344 | 0.068312 |
| C32 H47 N11 O4 | -0.49468 | 0.007449 | 0.068312 |
| 1509.1073@13.17305 | -0.49443 | 0.0074838 | 0.068449 |
| C30 H61 N21 O | -0.49391 | 0.0075577 | 0.068942 |
| C27 H47 N25 O | -0.49353 | 0.0076106 | 0.069241 |
| C21 H3 N3 O14 S5 | 0.49305 | 0.0076787 | 0.069592 |
| 418.7973@10.988921 | -0.49297 | 0.0076897 | 0.069592 |
| 773.6116@13.7109995 | 0.49236 | 0.0077776 | 0.070202 |
| 470.8043@11.0945015 | -0.49211 | 0.0078133 | 0.07021 |
| C15 H38 N14 O3 | -0.49207 | 0.0078193 | 0.07021 |
| 1132.3134@14.398666 | -0.4917 | 0.0078729 | 0.070455 |
| C20 H43 N O11 | -0.4916 | 0.0078875 | 0.070455 |
| C18 H33 N3 O | 0.4914 | 0.0079172 | 0.070536 |
| 493.5649@11.947277 | -0.49061 | 0.0080338 | 0.071266 |
| C23 H39 N3 O | -0.49056 | 0.0080405 | 0.071266 |
| C16 H35 N5 O S | -0.48905 | 0.0082665 | 0.073081 |
| C11 H19 N O | 0.4883 | 0.0083814 | 0.073898 |
| C19 H46 Cl N15 O | -0.48808 | 0.0084157 | 0.073898 |
| 570.6769@8.436177 | -0.48802 | 0.0084234 | 0.073898 |
| 783.491@12.6504 | -0.48748 | 0.008507 | 0.074442 |
| C23 H46 Cl N15 | -0.48731 | 0.0085345 | 0.074493 |
| C32 H50 O3 S2 | -0.48697 | 0.008588 | 0.07477 |
| C14 H26 N8 O3 + 5.9993935 | -0.4866 | 0.0086451 | 0.075077 |
| C16 H37 N9 O5 | -0.48646 | 0.0086675 | 0.075082 |
| 1567.0781@13.099728 | -0.48581 | 0.0087709 | 0.075608 |
| C39 H65 Cl N2 | -0.4858 | 0.0087722 | 0.075608 |
| C28 H52 O S2 | -0.48525 | 0.0088607 | 0.07618 |
| 833.6015@11.389999 | -0.48488 | 0.0089192 | 0.076491 |
| 793.5179@11.174819 | -0.48464 | 0.0089584 | 0.07663 |
| 1211.3737@13.574666 | 0.48443 | 0.008992 | 0.07663 |
| 634.7059@8.247894 | -0.48437 | 0.0090022 | 0.07663 |
| C45 H94 N2 O | -0.48379 | 0.0090964 | 0.077241 |
| 759.9539@8.133107 | -0.48201 | 0.009394 | 0.079527 |
| C18 O20 S | -0.48179 | 0.0094311 | 0.079527 |
| C25 H10 N2 O23 S | -0.48177 | 0.009435 | 0.079527 |
| C26 H53 Cl N2 O10 | -0.48139 | 0.009499 | 0.079863 |
| 435.762@11.0564995 | 0.4811 | 0.0095485 | 0.079863 |
| 479.7854@11.087 | 0.48061 | 0.0096333 | 0.079863 |
| 56.0691@11.9415 | 0.48058 | 0.0096382 | 0.079863 |
| C16 H40 N10 O7 | 0.48051 | 0.0096503 | 0.079863 |
| C28 H58 N18 O2 | -0.48026 | 0.0096928 | 0.079863 |
| 492.7848@11.1105 | 0.48025 | 0.0096952 | 0.079863 |
| C19 H45 N13 O8 | -0.48012 | 0.0097178 | 0.079863 |
| 1198.6552@7.4044657 | -0.48009 | 0.0097229 | 0.079863 |
| 648.7132@8.308463 | -0.48002 | 0.0097344 | 0.079863 |
| 525.7864@11.127001 | 0.47996 | 0.0097444 | 0.079863 |
| C22 H49 N23 O5 | -0.47991 | 0.0097536 | 0.079863 |
| 661.3748@11.089001 | 0.47969 | 0.0097915 | 0.079984 |
| 781.4648@8.210608 | -0.47944 | 0.0098353 | 0.080151 |
| 871.7477@14.749001 | 0.47904 | 0.0099064 | 0.080539 |
| C6 H6 N4 O2 | 0.4789 | 0.009931 | 0.080549 |
| C37 H43 N5 O6 | -0.4787 | 0.0099669 | 0.080649 |
| C33 H43 N7 S | -0.47776 | 0.010135 | 0.081815 |
| C29 H51 N13 S2 | -0.47622 | 0.010414 | 0.083704 |
| C30 H61 N9 O3 | -0.47615 | 0.010428 | 0.083704 |
| 785.503@12.826644 | -0.47601 | 0.010454 | 0.083704 |
| 759.452@8.1335335 | -0.47584 | 0.010485 | 0.083704 |
| C29 H44 N2 O4 | 0.47578 | 0.010496 | 0.083704 |
| C43 H69 N5 O S2 | -0.47568 | 0.010515 | 0.083704 |
| 1593.1213@13.165127 | 0.47541 | 0.010565 | 0.083913 |
| C21 H51 N13 O7 S | -0.47503 | 0.010636 | 0.084282 |
| C17 H36 N4 O S | 0.47466 | 0.010704 | 0.084623 |
| 465.528@8.629571 | 0.47424 | 0.010784 | 0.085059 |
| 831.5933@13.327541 | 0.47386 | 0.010857 | 0.08544 |
| C30 H53 N15 O7 | -0.4732 | 0.010983 | 0.086236 |
| C34 H22 N8 O2 S4 | -0.47301 | 0.011019 | 0.086279 |
| 84.1087@12.6017275 | -0.47291 | 0.011039 | 0.086279 |
| C24 H4 N4 O23 | -0.47249 | 0.011121 | 0.086723 |
| 583.8516@7.2865715 | -0.47174 | 0.011266 | 0.087654 |
| C37 H40 N4 O6 | -0.47141 | 0.011331 | 0.087964 |
| C15 H38 Cl N13 O4 | -0.47089 | 0.011435 | 0.08849 |
| 913.5445@8.649 | -0.47073 | 0.011466 | 0.08849 |
| 1567.1173@13.15 | 0.47067 | 0.011478 | 0.08849 |
| C20 H10 N2 O22 S2 | -0.47055 | 0.011502 | 0.08849 |
| C28 H51 N15 O6 | -0.46978 | 0.011657 | 0.089484 |
| C21 H41 N27 | -0.46905 | 0.011806 | 0.090425 |
| C21 H4 O26 | -0.46875 | 0.011868 | 0.090533 |
| 574.834@8.7635 | -0.46872 | 0.011873 | 0.090533 |
| C29 H10 N2 O19 | 0.46829 | 0.011961 | 0.091005 |
| 805.9571@8.284154 | -0.46788 | 0.012048 | 0.091463 |
| 768.4123@7.347733 | 0.46678 | 0.012277 | 0.092994 |
| 781.7184@9.111 | -0.46603 | 0.012437 | 0.093806 |
| 1590.0977@13.126749 | 0.46602 | 0.012438 | 0.093806 |
| C20 H36 N2 S | -0.46564 | 0.012521 | 0.09422 |
| C15 H12 O18 S | -0.46526 | 0.012602 | 0.094437 |
| C26 H2 O19 S | -0.46525 | 0.012604 | 0.094437 |
| 797.5322@12.9182005 | -0.46343 | 0.013002 | 0.097207 |
| C19 H39 N O2 | -0.46236 | 0.013241 | 0.098779 |
| 530.8171@7.491895 | -0.46206 | 0.013309 | 0.099066 |
| 1127.7795@11.197499 | -0.46131 | 0.013479 | 0.10011 |
| 454.5204@8.5718 | 0.46082 | 0.01359 | 0.10073 |
| C20 H51 N19 O6 S | -0.46058 | 0.013645 | 0.10091 |
| 779.536@11.169501 | -0.45956 | 0.013881 | 0.10244 |
| C31 H47 N O | -0.45923 | 0.013959 | 0.10249 |
| 783.9416@8.209999 | -0.45918 | 0.013971 | 0.10249 |
| C32 H59 N5 O4 S | -0.45915 | 0.013978 | 0.10249 |
| C21 H2 O26 | -0.45893 | 0.014031 | 0.10266 |
| C17 H2 O10 S5 | -0.45876 | 0.014071 | 0.10274 |
| C43 H84 Cl N3 O2 | -0.45852 | 0.014128 | 0.10293 |
| C19 H45 Cl N18 O S | -0.45763 | 0.014339 | 0.10425 |
| C41 H68 O7 S | 0.456 | 0.014735 | 0.10669 |
| C46 H92 N2 | -0.456 | 0.014736 | 0.10669 |
| C19 H N O7 S5 | -0.45541 | 0.014882 | 0.10752 |
| Cortisol 21-acetate | -0.45528 | 0.014913 | 0.10752 |
| 384.2006@7.3542504 | -0.45455 | 0.015095 | 0.1086 |
| C30 H29 N3 O6 S5 | -0.45438 | 0.015139 | 0.10869 |
| 396.3639@12.482241 | -0.45424 | 0.015172 | 0.1087 |
| 541.6578@8.291108 | -0.4541 | 0.015209 | 0.10874 |
| C21 H45 N5 O8 | -0.45334 | 0.015401 | 0.10988 |
| C34 H73 N3 O3 S5 | -0.45312 | 0.015458 | 0.11006 |
| C17 H43 N13 O5 S | -0.4521 | 0.015721 | 0.1117 |
| C20 H33 Cl N16 O5 | -0.45183 | 0.015789 | 0.11196 |
| C17 H17 N O19 S | -0.45156 | 0.015861 | 0.11224 |
| C17 H6 O9 S5 | -0.45137 | 0.015911 | 0.11236 |
| 523.8466@11.150875 | -0.45047 | 0.016149 | 0.1138 |
| 781.9666@8.210929 | -0.44902 | 0.016535 | 0.11629 |
| C36 H36 Cl2 O8 | -0.44881 | 0.016593 | 0.11646 |
| C41 H8 N6 O5 | -0.44867 | 0.016631 | 0.11649 |
| C8 H19 N7 O2 | 0.4476 | 0.016923 | 0.11817 |
| C22 H51 N5 S2 | -0.44754 | 0.01694 | 0.11817 |
| 984.3676@12.9855 | -0.44688 | 0.017121 | 0.11905 |
| C24 H47 Cl N4 O2 | -0.44683 | 0.017136 | 0.11905 |
| C6 H13 Cl3 N4 O5 | -0.44652 | 0.017223 | 0.11938 |
| 831.5826@13.348001 | -0.44631 | 0.017281 | 0.11938 |
| 1011.3673@12.999667 | -0.44629 | 0.017287 | 0.11938 |
| C11 H26 N6 O S | 0.4461 | 0.01734 | 0.1195 |
| 850.3639@4.8205004 | 0.44586 | 0.017408 | 0.11973 |
| C12 H6 Cl3 N O4 S5 | 0.44534 | 0.017556 | 0.12051 |
| 923.7996@15.029429 | -0.44487 | 0.017688 | 0.12118 |
| 782.5693@11.987624 | -0.44462 | 0.017761 | 0.12143 |
| 1181.8632@13.575667 | 0.44405 | 0.017924 | 0.12231 |
| 419.7838@8.056572 | -0.44356 | 0.018065 | 0.12264 |
| 727.5587@12.458667 | -0.44353 | 0.018074 | 0.12264 |
| 607.8428@7.29275 | -0.44351 | 0.01808 | 0.12264 |
| 281.2605@11.293848 | -0.44312 | 0.018196 | 0.12318 |
| C26 H11 N11 O3 S | -0.44278 | 0.018295 | 0.1236 |
| C17 H16 O19 S | -0.44256 | 0.01836 | 0.1236 |
| 108.09@6.7783475 | -0.44254 | 0.018366 | 0.1236 |
| C36 H67 N O5 S3 | -0.44221 | 0.018461 | 0.124 |
| 420.7409@6.142466 | -0.44174 | 0.018601 | 0.12461 |
| 759.4485@9.058429 | -0.44167 | 0.018624 | 0.12461 |
| 840.1663@8.9914 | -0.44106 | 0.018806 | 0.12558 |
| 208.1734@8.6395 | 0.43954 | 0.019267 | 0.12826 |
| 1529.0444@13.050942 | -0.43949 | 0.019281 | 0.12826 |
| C15 H40 N14 O4 S | -0.43889 | 0.019465 | 0.12923 |
| 229.246@8.035 | 0.4386 | 0.019555 | 0.12958 |
| 981.3801@12.96375 | -0.4384 | 0.019619 | 0.12975 |
| C17 H44 N14 O5 S | -0.43823 | 0.019672 | 0.12985 |
| 803.3715@7.3702307 | -0.43471 | 0.020793 | 0.13699 |
| 716.7493@8.589714 | -0.43458 | 0.020837 | 0.13701 |
| 516.8406@10.884167 | -0.43425 | 0.020944 | 0.13746 |
| 897.6027@11.063816 | -0.43411 | 0.020993 | 0.13751 |
| 759.7011@9.0476675 | -0.43371 | 0.021123 | 0.1381 |
| 803.9801@8.285571 | -0.4332 | 0.021293 | 0.13895 |
| C25 H46 O S2 | 0.43302 | 0.021351 | 0.13907 |
| C22 H54 N22 O2 S2 | -0.43236 | 0.021573 | 0.14025 |
| 964.5624@8.2915 | -0.4319 | 0.021729 | 0.14099 |
| C18 H41 Cl2 N5 | -0.43144 | 0.021883 | 0.14172 |
| 561.837@7.166107 | -0.43122 | 0.021959 | 0.14186 |
| C14 H37 N11 O8 | -0.43114 | 0.021986 | 0.14186 |
| 797.4617@5.92525 | -0.431 | 0.022036 | 0.14191 |
| C32 H52 N8 O4 | -0.43068 | 0.022144 | 0.14201 |
| C15 H34 N6 | -0.43064 | 0.022159 | 0.14201 |
| 1017.5939@6.874778 | -0.43048 | 0.022212 | 0.14201 |
| 373.4432@4.9967504 | -0.43042 | 0.022233 | 0.14201 |
| 815.5492@13.085166 | -0.43031 | 0.02227 | 0.14201 |
| C19 H43 N7 O6 S | -0.43023 | 0.022298 | 0.14201 |
| C14 H34 N10 O4 | 0.42992 | 0.022408 | 0.14244 |
| C26 H47 N15 O5 | -0.42969 | 0.022488 | 0.14269 |
| C18 H38 N8 O4 S | -0.42946 | 0.022565 | 0.14286 |
| 527.7363@9.514185 | -0.42937 | 0.022599 | 0.14286 |
| C15 H22 O S3 | 0.42915 | 0.022675 | 0.14308 |
| C37 H80 N4 S | -0.42876 | 0.022811 | 0.14368 |
| 421.7634@13.292 | 0.42862 | 0.02286 | 0.14372 |
| C33 H27 N9 O3 | 0.42841 | 0.022935 | 0.14393 |
| C17 H4 N2 O16 | -0.42828 | 0.022982 | 0.14396 |
| C26 H8 N2 O22 | -0.42801 | 0.023078 | 0.14429 |
| 641.2035@12.484269 | -0.42778 | 0.023159 | 0.14429 |
| 526.6485@8.211465 | -0.42778 | 0.023161 | 0.14429 |
| 110.1074@3.3285007 | 0.42725 | 0.023348 | 0.1452 |
| 771.6282@13.819858 | -0.42589 | 0.023839 | 0.14799 |
| C16 H39 N15 O2 | 0.42569 | 0.023913 | 0.14817 |
| C21 H43 N11 O7 | -0.42553 | 0.023974 | 0.14828 |
| C12 H3 N O15 S | -0.42507 | 0.024142 | 0.14894 |
| 863.6162@11.020192 | -0.425 | 0.024167 | 0.14894 |
| C35 H14 Cl N O10 | -0.42433 | 0.024417 | 0.15001 |
| 965.0636@8.303249 | -0.4243 | 0.024428 | 0.15001 |
| C21 H49 N17 O7 | -0.42398 | 0.024547 | 0.15048 |
| 847.5069@8.435072 | -0.42363 | 0.024679 | 0.15102 |
| 454.3944@12.9447775 | -0.42305 | 0.024895 | 0.15192 |
| C34 H67 N9 O2 S2 | 0.423 | 0.024915 | 0.15192 |
| C19 H40 N6 O5 S | -0.42257 | 0.025078 | 0.15264 |
| 795.615@13.785142 | -0.42243 | 0.025133 | 0.15271 |
| 809.5496@10.998002 | -0.42181 | 0.025367 | 0.15385 |
| C14 H31 N O | -0.42146 | 0.025502 | 0.1544 |
| C16 H43 N11 O3 | -0.42114 | 0.025628 | 0.15489 |
| N-Acetyl-L-Histidine | 0.42088 | 0.025725 | 0.15521 |
| 546.573@8.618333 | -0.42049 | 0.025881 | 0.15587 |
| C18 H35 N11 O S | -0.41998 | 0.026079 | 0.15658 |
| C31 O20 | -0.41995 | 0.02609 | 0.15658 |
| C21 H43 N7 O2 S | -0.41948 | 0.026274 | 0.15742 |
| 801.5548@13.023407 | -0.41897 | 0.026478 | 0.15836 |
| 783.5952@13.447523 | -0.41838 | 0.026713 | 0.15949 |
| 495.7949@6.7097864 | -0.41812 | 0.026818 | 0.15963 |
| 530.8097@7.0232854 | -0.41799 | 0.02687 | 0.15963 |
| C25 Cl N S5 | -0.41798 | 0.026875 | 0.15963 |
| C18 H2 O17 S3 | -0.41766 | 0.027001 | 0.15972 |
| C33 H48 O | -0.41763 | 0.027015 | 0.15972 |
| C14 H31 N3 O2 S2 | -0.41751 | 0.027063 | 0.15972 |
| C8 H18 N14 | 0.41746 | 0.027085 | 0.15972 |
| C17 H41 N13 O7 | -0.41736 | 0.027123 | 0.15972 |
| C18 H43 N7 O10 | -0.41719 | 0.027193 | 0.15986 |
| C11 H4 O16 S | -0.41706 | 0.027246 | 0.15989 |
| C14 H4 N4 O13 S | -0.41669 | 0.027397 | 0.1603 |
| C28 H44 N4 S | -0.41666 | 0.027409 | 0.1603 |
| C8 H18 O5 | 0.41635 | 0.027536 | 0.16077 |
| C31 H68 Cl N5 O10 | -0.41584 | 0.027745 | 0.16172 |
| C19 H13 N5 O9 S3 | -0.41567 | 0.027815 | 0.16185 |
| C17 H40 N8 O5 S | -0.41526 | 0.027987 | 0.16253 |
| ?-Estradiol + 7.9324293 | 0.41516 | 0.028026 | 0.16253 |
| 786.567@11.946625 | -0.41499 | 0.028098 | 0.16267 |
| 283.278@11.724875 | -0.41478 | 0.028185 | 0.16291 |
| 784.5624@13.1614 | -0.4145 | 0.028303 | 0.16331 |
| 241.2692@7.8270836 | 0.41398 | 0.028521 | 0.16428 |
| C13 H16 O | 0.41387 | 0.028567 | 0.16428 |
| 528.6357@8.2177 | -0.41368 | 0.02865 | 0.16449 |
| 852.5675@13.0415 | 0.41337 | 0.028783 | 0.16497 |
| 914.0486@8.641999 | -0.413 | 0.028941 | 0.1656 |
| 775.2934@12.653999 | 0.41248 | 0.02916 | 0.16658 |
| 1278.3512@15.065429 | -0.41215 | 0.029302 | 0.16712 |
| 7-ketocholesterol + 12.597 | 0.41199 | 0.029374 | 0.16725 |
| 868.5655@11.024223 | -0.41172 | 0.02949 | 0.16763 |
| 811.5366@13.005833 | -0.4116 | 0.029543 | 0.16763 |
| C23 H20 N18 O S2 | -0.4114 | 0.029627 | 0.16763 |
| C13 H9 N O17 S | -0.41138 | 0.029637 | 0.16763 |
| C32 H53 N11 O4 | -0.41118 | 0.029724 | 0.16785 |
| 883.5853@10.764593 | -0.41076 | 0.029908 | 0.16861 |
| C18 H43 N13 O5 S | -0.41055 | 0.03 | 0.16885 |
| 474.8317@8.18775 | -0.41043 | 0.030052 | 0.16887 |
| C35 H40 N2 O | 0.41031 | 0.030106 | 0.1689 |
| C14 H37 N15 O3 S | -0.41011 | 0.030193 | 0.16911 |
| MG(18:1(9Z)/0:0/0:0)[rac] | -0.40976 | 0.030349 | 0.16971 |
| 824.8213@8.931 | -0.4096 | 0.030421 | 0.16983 |
| 539.8228@7.026536 | -0.409 | 0.03069 | 0.17089 |
| C16 H11 N7 | 0.40895 | 0.030709 | 0.17089 |
| C25 H44 N16 O | 0.40824 | 0.03103 | 0.17239 |
| C38 H78 N6 O | -0.408 | 0.03114 | 0.17273 |
| C28 H48 N2 O | -0.4067 | 0.031732 | 0.17541 |
| C22 H31 N3 O | -0.40666 | 0.031754 | 0.17541 |
| 1223.8643@13.540201 | 0.40657 | 0.031792 | 0.17541 |
| C42 H83 N O9 | -0.40649 | 0.031828 | 0.17541 |
| C34 H73 N7 | -0.40629 | 0.031923 | 0.17565 |
| 2-Hexyldecanoic acid | 0.40618 | 0.031974 | 0.17565 |
| 442.7608@6.8532495 | -0.40577 | 0.032164 | 0.17641 |
| 552.8212@7.164214 | -0.4049 | 0.03257 | 0.17835 |
| C28 H61 Cl N4 O13 | -0.40428 | 0.032861 | 0.17966 |
| 775.5641@10.951072 | -0.40406 | 0.032969 | 0.17997 |
| C18 H29 N3 O | -0.40346 | 0.033251 | 0.18117 |
| 523.8089@8.510624 | -0.40337 | 0.033295 | 0.18117 |
| 751.4016@9.536858 | -0.40319 | 0.033384 | 0.18137 |
| 765.5235@10.961518 | -0.40294 | 0.0335 | 0.18172 |
| C18 H43 N9 O | -0.4025 | 0.033713 | 0.18258 |
| 644.7216@8.764923 | -0.40234 | 0.033792 | 0.18272 |
| C33 H4 O21 | -0.40222 | 0.03385 | 0.18275 |
| 441.7882@12.894333 | -0.402 | 0.033956 | 0.18303 |
| C20 H21 N7 | -0.40171 | 0.034096 | 0.1835 |
| C17 H38 N2 O6 | -0.40118 | 0.034359 | 0.18447 |
| C28 H60 N16 O5 | -0.40113 | 0.034383 | 0.18447 |
| 408.3407@12.855 | -0.40083 | 0.034529 | 0.18496 |
| C25 H49 N11 O9 | -0.40069 | 0.034596 | 0.18503 |
| 1280.3448@15.0581665 | -0.40018 | 0.034852 | 0.18611 |
| 420.748@6.466625 | -0.39978 | 0.035047 | 0.18686 |
| 189.1329@0.9966666 | 0.39913 | 0.035373 | 0.18807 |
| C13 H34 N10 O7 + 6.8533564 | -0.39909 | 0.035393 | 0.18807 |
| C11 H2 Cl N O11 S4 | -0.39892 | 0.03548 | 0.18807 |
| 801.4589@8.156375 | -0.39889 | 0.035493 | 0.18807 |
| 795.4707@8.837249 | -0.39879 | 0.035547 | 0.18807 |
| C33 H54 N2 O S2 | 0.39802 | 0.035936 | 0.18966 |
| 552.8226@8.667 | -0.39794 | 0.035976 | 0.18966 |
| 457.8059@11.0650015 | -0.39787 | 0.036012 | 0.18966 |
| C26 H39 N3 O3 | -0.39748 | 0.036213 | 0.19042 |
| 874.1937@10.042062 | 0.39638 | 0.036776 | 0.19309 |
| C15 H25 N O S3 | 0.39605 | 0.036949 | 0.19358 |
| 438.7872@12.833799 | -0.39598 | 0.036983 | 0.19358 |
| Jasmonic acid + 6.6657147 | -0.39524 | 0.037373 | 0.19533 |
| C30 H37 N7 O2 | -0.39513 | 0.03743 | 0.19533 |
| 4-acetamidobutanoate + 0.91700006 | 0.39408 | 0.037984 | 0.1979 |
| C17 H41 N5 O S3 | -0.39388 | 0.038092 | 0.1979 |
| C15 H36 N8 O4 S | -0.39387 | 0.038095 | 0.1979 |
| C16 H34 N14 O4 | -0.3937 | 0.038188 | 0.19808 |
| Jasmonic acid | -0.39349 | 0.038301 | 0.19831 |
| 1211.3037@13.099667 | 0.39336 | 0.038368 | 0.19831 |
| C13 H25 N S2 | 0.39329 | 0.038405 | 0.19831 |
| 1188.3158@13.185999 | 0.39314 | 0.038487 | 0.19843 |
| C28 H33 N7 O2 | -0.3929 | 0.038614 | 0.19879 |
| C17 H38 N4 O4 | 0.39235 | 0.038913 | 0.20003 |
| 757.5935@13.368693 | -0.39185 | 0.039186 | 0.20106 |
| 1043.3281@12.977251 | -0.39177 | 0.03923 | 0.20106 |
| C27 H44 O4 | -0.39123 | 0.039525 | 0.20219 |
| C11 H35 Cl N12 S | -0.39115 | 0.039567 | 0.20219 |
| C45 H81 N O S | -0.39092 | 0.039693 | 0.20253 |
| 494.4291@12.977112 | -0.3907 | 0.039816 | 0.20272 |
| 1247.7993@13.080999 | 0.39064 | 0.039849 | 0.20272 |
| 986.9785@13.092751 | -0.38874 | 0.040911 | 0.20782 |
| C28 H54 N2 S3 | 0.38845 | 0.041077 | 0.20835 |
| 751.5041@10.635038 | -0.38796 | 0.04135 | 0.20925 |
| (Methylthio)acetic acid | 0.38792 | 0.041376 | 0.20925 |
| C6 H16 N2 O S | -0.38733 | 0.04171 | 0.21063 |
| C27 H53 N5 O10 | -0.38709 | 0.041848 | 0.21102 |
| 448.7885@11.06563 | -0.3869 | 0.041961 | 0.21126 |
| C12 H30 N18 | -0.38676 | 0.042037 | 0.21126 |
| C38 H67 N S2 | -0.38669 | 0.042079 | 0.21126 |
| C19 H3 N3 O15 S2 | 0.38628 | 0.042318 | 0.21215 |
| 825.4919@8.364161 | -0.38612 | 0.042412 | 0.21231 |
| C19 H44 N10 O | -0.38568 | 0.042669 | 0.21328 |
| C18 H45 N21 S2 | -0.3855 | 0.042771 | 0.21348 |
| C31 H40 N18 O2 | 0.38494 | 0.043098 | 0.2148 |
| C12 H24 N2 O3 | 0.38368 | 0.043847 | 0.21822 |
| 757.2257@13.450334 | -0.38277 | 0.044389 | 0.22026 |
| 459.7822@11.0664015 | -0.3827 | 0.044435 | 0.22026 |
| C8 H12 | 0.38267 | 0.044449 | 0.22026 |
| 521.5856@12.166408 | -0.38244 | 0.044591 | 0.22039 |
| C44 O7 S2 | -0.38221 | 0.044728 | 0.22039 |
| 544.6484@8.347251 | -0.3821 | 0.044796 | 0.22039 |
| 558.8673@11.199376 | -0.38208 | 0.044806 | 0.22039 |
| C36 H37 Cl O8 | -0.38202 | 0.044845 | 0.22039 |
| C26 H25 N13 O12 | -0.38199 | 0.04486 | 0.22039 |
| 1238.3202@13.208 | 0.38173 | 0.04502 | 0.22086 |
| 679.143@10.896999 | -0.38148 | 0.045172 | 0.22129 |
| C22 H49 N7 O12 | -0.38133 | 0.045265 | 0.22136 |
| 1589.0319@12.96675 | -0.38125 | 0.045315 | 0.22136 |
| 863.2723@13.222444 | -0.38105 | 0.045433 | 0.22162 |
| 794.9619@8.2792 | 0.38042 | 0.045821 | 0.22296 |
| 1596.1149@13.243 | 0.38023 | 0.045938 | 0.22296 |
| 760.5965@13.688 | 0.38022 | 0.045946 | 0.22296 |
| 715.2495@10.025 | 0.38019 | 0.045967 | 0.22296 |
| C8 H9 N S | 0.37985 | 0.046175 | 0.22366 |
| C34 H60 N6 O2 S | -0.37817 | 0.047232 | 0.22845 |
| 991.7215@13.0355 | -0.37682 | 0.048087 | 0.23172 |
| C22 H37 N9 O4 | -0.37675 | 0.048134 | 0.23172 |
| C27 H56 N2 | -0.37667 | 0.048186 | 0.23172 |
| C16 O15 S2 | -0.37662 | 0.048219 | 0.23172 |
| C29 H57 N11 O11 + 6.23825 | -0.37658 | 0.048244 | 0.23172 |
| 831.6037@13.660667 | -0.37644 | 0.048334 | 0.23183 |
| 428.3643@12.8455 | -0.37604 | 0.048589 | 0.23273 |
| 1180.826@13.1933 | 0.37566 | 0.048838 | 0.23359 |
| 435.7932@11.033884 | -0.37507 | 0.049221 | 0.2351 |

P value after Spearman correlation analyses. FDR: False-discovery corrected p value after Benjamini-Hochberg correction. * ions presented are preliminary characterized by potential elemental formulae or, when it is not possible, characterized by m/z and retention time (in minutes) separated by the @ symbol.
